# Single-cell profiling of brain pericyte heterogeneity following ischemic stroke unveils distinct pericyte subtype-targeted neural reprogramming potential and its underlying mechanisms

**DOI:** 10.7150/thno.97165

**Published:** 2024-09-23

**Authors:** Allison Loan, Nidaa Awaja, Margarita Lui, Charvi Syal, Yiren Sun, Sailendra N Sarma, Ragav Chona, William B Johnston, Alex Cordova, Devansh Saraf, Anabella Nakhlé, Kaela O'Connor, Jacob Thomas, Joseph Leung, Matthew Seegobin, Ling He, Fredric E Wondisford, David J Picketts, Eve C Tsai, Hing Man Chan, Jing Wang

**Affiliations:** 1 Regenerative Medicine Program, Ottawa Hospital Research Institute, Ottawa, ON, K1H 8L6, Canada.; 2 Department of Biology, Faculty of Science, University of Ottawa, Ottawa, ON, K1H 8M5, Canada.; 3 Neuroscience Program, Ottawa Hospital Research Institute, Ottawa, ON, K1H 8L6, Canada.; 4 Current address: National Wildlife Research Center, Environment and Climate Change Canada, Ottawa, ON, K1S 5B6, Canada.; 5 Department of Cellular and Molecular Medicine, Faculty of Medicine, University of Ottawa, Ottawa, ON, K1H 8M5, Canada.; 6 Current address: Children's Hospital of Eastern Ontario Research Institute, Ottawa, ON K1H 8L1, Canada.; 7 Current address: Department of Cellular and Molecular Medicine, University of Ottawa, Ottawa, ON K1H 8M5, Canada.; 8 Current address: Program in Neuroscience and Mental Health, SickKids Research Institute, Toronto, Ontario M5G 1L7, Canada.; 9 Departments of Basic Medical Sciences and Internal Medicine, University of Arizona College of Medicine-Phoenix, Phoenix, AZ, 85004, USA.; 10 Department of Biochemistry, Microbiology and Immunology, Faculty of Medicine, University of Ottawa, Ottawa, ON, K1H 8M5, Canada.; 11 University of Ottawa Brain and Mind Research Institute, Ottawa, ON, K1H 8M5, Canada.; 12 Department of Surgery, Faculty of Medicine, University of Ottawa, Ottawa, ON, K1H 8M5, Canada.; 13 Canadian Partnership for Stroke Recovery, Ottawa, ON, K1G 5Z3, Canada.

**Keywords:** CBP S436 phosphorylation, focal ischemic stroke, induced neural stem cells, pericytes, cellular reprogramming, neuronal differentiation, acetylation, Sox2, histone 2B

## Abstract

**Rationale:** Brain pericytes can acquire multipotency to produce multi-lineage cells following injury. However, pericytes are a heterogenous population and it remains unknown whether there are different potencies from different subsets of pericytes in response to injury.

**Methods:** We used an ischemic stroke model combined with pericyte lineage tracing animal models to investigate brain pericyte heterogeneity under both naïve and brain injury conditions via single-cell RNA-sequencing and immunohistochemistry analysis. In addition, we developed an NG2^+^ pericyte neural reprogramming culture model from both murine and humans to unveil the role of energy sensor, AMP-dependent kinase (AMPK), activity in modulating the reprogramming/differentiation process to convert pericytes to functional neurons by targeting a Ser 436 phosphorylation on CREB-binding protein (CBP), a histone acetyltransferase.

**Results:** We showed that two distinct pericyte subpopulations, marked by NG2^+^ and Tbx18^+^, had different potency following brain injury. NG2^+^ pericytes expressed dominant neural reprogramming potential to produce newborn neurons, while Tbx18^+^ pericytes displayed dominant multipotency to produce endothelial cells, fibroblasts, and microglia following ischemic stroke. In addition, we discovered that AMPK modulators facilitated pericyte-to-neuron conversion by modulating Ser436 phosphorylation status of CBP, to coordinate an acetylation shift between Sox2 and histone H2B, and to regulate Sox2 nuclear-cytoplasmic trafficking during the reprogramming/differentiation process. Finally, we showed that sequential treatment of compound C (CpdC) and metformin, AMPK inhibitor and activator respectively, robustly facilitated the conversion of human pericytes into functional neurons.

**Conclusion:** We revealed that two distinct subtypes of pericytes possess different reprogramming potencies in response to physical and ischemic injuries. We also developed a genomic integration-free methodology to reprogram human pericytes into functional neurons by targeting NG2^+^ pericytes.

## Introduction

Brain pericytes are part of mural cells that wrap around microvessels, such as arterioles, venules, and capillaries to form a major component of the blood-brain barrier and play an important role in angiogenesis and vessel stabilization 1. Brain pericytes are a heterogeneous population and present with morphological diversity ranging from large ensheathing pericytes to smaller mesh and thin-strand pericytes 2. Pericytes also arise from diverse origins such as neural crest or mesenchyme 3,4, and present various marker genes including neuron-glial antigen 2 (NG2), platelet-derived growth factor receptor β (Pdgfrβ), Rgs5, and Tbx18, each of which is commonly used for lineage tracing 5. It remains unknown how much overlap in cell composition these lineage-traced pericytes share under naïve conditions and in response to injury.

One unique feature of brain pericytes is their plasticity in response to injury. In response to a stab injury or an ischemic stroke-related injury, brain pericytes can give rise to a variety of cell types, including endothelial cells 6,7, fibroblasts 8,9, microglia 10,11, and neural stem cells 10,12,13. Brain pericyte-derived endothelial cells, fibroblasts and microglia following ischemic stroke have all been confirmed with genetic fate mapping using either *Tbx18-CreER^T2^/Rosa26-tdTomato*, *Glast-CreER^T2^/Rosa26-tdTomato*, or *Rgs5-YFP* mouse lines 11,14,15. Although there are no direct lineage tracing results to show the neurogenic potential of brain pericytes, we and other research groups have cultured pericytes isolated from ischemic-injured brain cortical tissues and showed that they have the capability to form induced-neurospheres (i-neurospheres) upon exposure to neural conditioned medium 7,16. In addition, forebrain pericytes originate from embryonic neural crest cells, which feature both neuroectodermal and mesenchymal cell properties 17. This suggests that brain injury, especially ischemic stroke-related injury, can readily induce the reprogramming of cortical pericytes into neural lineage cells. Thus, it is important to identify which pericyte subtypes in the cerebral cortex possess neurogenic potential in response to ischemic brain injury. This will be the foundation for the development of targeted cell therapy to enhance local neurogenesis at the site of stroke-related injury from a specific subtype of pericytes without imperiling other physiological functions of pericytes.

Tbx18 expressing pericytes lineage-traced with the *Tbx18-CreER^T2^/Rosa26-tdTomato* in a mouse model revealed that they do not produce neural cells and fibrotic tissues in the cortex following cortical stab injury 18. On the other hand, recent single-cell RNA-sequencing (scRNA-seq) analysis of *Tbx18-tdT^+^* pericytes shows that Tbx18 expressing pericytes can produce fibroblasts, endothelial cells, and microglia following ischemic stroke by photothrombosis 14. In addition, NG2-expressing pericytes in the cerebral cortex can be traced using the *NG2-CreER^T2^/Rosa26-tdTomato* transgenic mouse model 19. However, *NG2-tdT^+^* is also expressed in oligodendrocyte precursors (OPCs) in the cerebral cortex with different morphology, location, and brightness than *NG2-tdT^+^* pericytes 19-21. Many studies have used the *NG2-CreER^T2^/Rosa26-tdTomato* transgenic mouse to study the function of either pericytes or OPCs, but not both types of cells at the same time 22-24. Although NG2^+^ glia cells are known to have the capability to produce neuronal lineage cells following brain injury, it remains unknown regarding the cell plasticity of NG2^+^ pericytes 25,26. Thus, to unravel the heterogeneity and cell plasticity of cortical pericytes in response to brain injury, scRNA-seq combined with pericyte genetic fate mapping in animal models provides a powerful tool to elucidate their underlying cellular/molecular mechanisms.

Recently, we cultured NG2^+^/Pdgfrβ^+^ pericytes isolated from ischemic injured cortices and were able to reprogram them into multipotent neural stem cells (NSCs) by forming i-neurospheres upon exposure to neural conditioned medium 16. Furthermore, we showed that inactivation of the atypical protein kinase C (aPKC)-mediated Ser436 phosphorylation in histone acetyltransferase CREB-binding protein (CBP) (aPKC-CBP pathway), using a phospho-null knock-in mouse strain (*Cbp*S436A) or a pharmacological inhibitor of AMP-dependent kinase (AMPK): compound C (CpdC). This robustly increased reprogramming efficiency of ischemia-activated pericytes (a-pericytes) into multipotent NSCs 16. In contrast, our early work showed that activation of the aPKC-CBP pathway, using a pharmacological activator of AMPK (metformin), promoted neuronal differentiation of adult neural stem and progenitor cells (NPCs) 27. Intriguingly, CBP is an acetyltransferase known to modify both histone and non-histone proteins 28-31. A previous report demonstrates that acetylation of Sox2 by CBP induces Sox2 nuclear export followed by its proteasomal degradation to promote differentiation of embryonic stem cells 28. Others show that deacetylation of Sox2 by Sirt1, a member of the sirtuin deacetylase family, is required for the somatic reprogramming of embryonic fibroblasts into induced pluripotent stem cells (iPSCs) 32. These studies prompted us to ask whether the aPKC-CBP pathway modulates both neural reprogramming of a-pericytes and neuronal differentiation through a common molecular mechanism, coordinating acetylation between Sox2 and histone 2B (H2B) to regulate Sox2 nuclear import/export.

Here, we used scRNA-seq and immunohistochemistry analysis to show that NG2^+^ cortical pericytes possess a strong neural reprogramming potential to produce local neurons at the site of lesion in response to ischemic injury, while the Tbx18^+^ pericyte subpopulation holds strong multipotency to produce endothelial cells, fibroblasts, and microglia with minimal neural reprogramming potency. In addition, we developed an NG2^+^ pericyte neural reprogramming culture model by modelling ischemic stroke *in vitro*. Subsequently, we discovered that modulators of the energy sensor AMPK facilitated pericyte-neuron conversion through modulating Ser436 phosphorylation status of CBP, which coordinates an acetylation shift between Sox2 and H2B and facilitates Sox2 nuclear-cytoplasmic trafficking for the reprogramming/differentiation process. Finally, we identified that sequential treatment of CpdC and metformin, AMPK inhibitor and activator respectively, robustly facilitated human pericytes to be reprogrammed into functional neurons.

## Results

### Naïve *NG2^+^* pericytes and *Tbx18^+^* pericytes are two distinct pericyte populations

**Figure 1:**
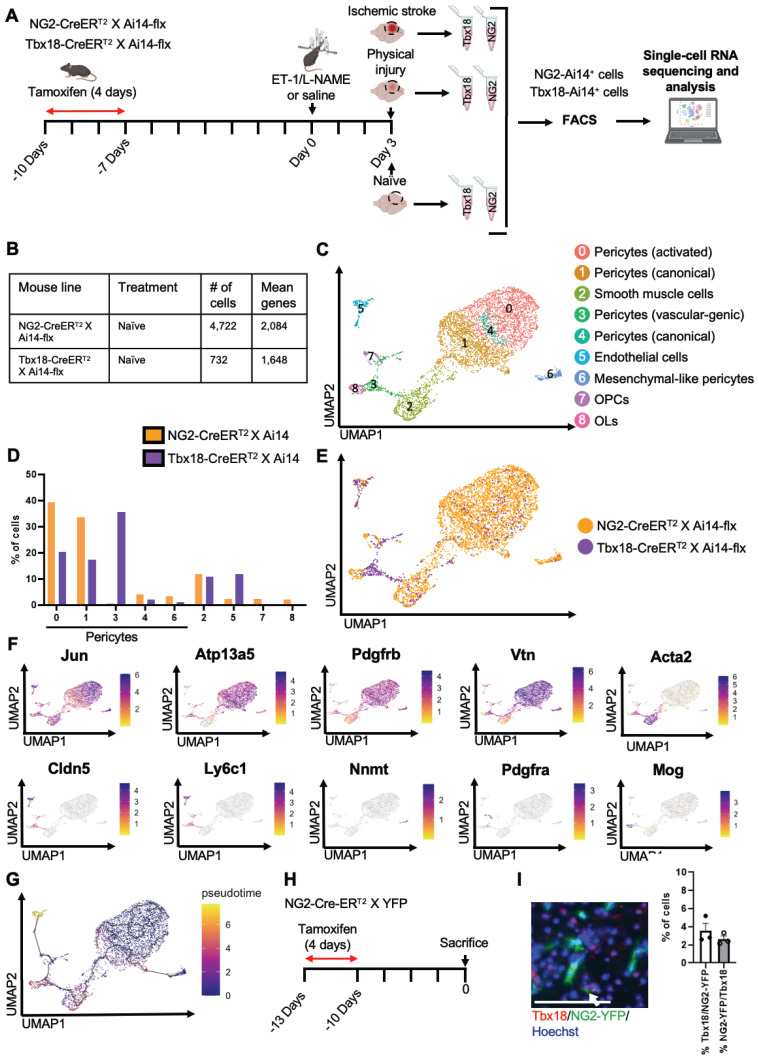
** Naïve *NG2^+^* pericytes and *Tbx18^+^* pericytes are two distinct pericyte populations.** (A) Schematic of experimental timeline, created with BioRender.com. 10-7 days before injury both *NG2-CreER^T2^/Ai14-flx* and *Tbx18-CreER^T2^/Ai14-flx* mice received tamoxifen injections daily for 4 days. Intracerebral injections of ET-1/L-NAME (or saline) were performed 3 days prior to scRNA-seq. Three groups of *Tbx18-Ai14^+^* and *NG2-Ai14^+^* cells from 1) no injury (naïve), 2) physical injury (saline), and 3) ischemic injury (ET-1/L-NAME) were FAC sorted for tdT (Ai14)^+^/DAPI^-^ and scRNA-seq was performed. (B) Number of cells obtained and mean number of genes per cell for both naïve *NG2-tdT^+^* and naïve *Tbx18-tdT^+^.* (C) Visualization of cells from naïve *NG2-tdT^+^* and naïve *Tbx18-tdT^+^* groups after PCA and UMAP, coloured by Seurat clustering and annotated by cell type. (D) Proportion of cells in each cluster for naïve *NG2-tdT^+^* and naïve *Tbx18-tdT^+^*. (E) UMAP visualization of *NG2-tdT^+^* (orange) and *Tbx18-tdT^+^* (purple) groups. (F) Visualization of the total cell population after PCA and UMAP, coloured by expression of key marker genes (*Jun*, *Atp13a5*, *Pdgfrβ*, *Vtn*, *Acta2*, *Cldn5*, *Ly6c1*, *Nnmt*, *Pdgfrα*, and *Mog*). (G) Visualization of the total cell population colored by pseudotime using Monocle3. (H) Flowchart of naïve *NG2-CreER^T2^ X YFP-flx* mice receiving tamoxifen treatment 10 days prior to sacrifice for immunohistochemistry. (I-J) Image and quantitative analysis of the proportion of Tbx18^+^/*NG2-YFP^+^* and *NG2-YFP^+^*/Tbx18^+^ cells in cortical sections from naïve mice, immunostained for *NG2-YFP* (green) and Tbx18 (red) and counterstained for Hoechst (blue). Scale bar: 50 µm.

To determine whether there were differences between *NG2-tdT^+^* pericytes and *Tbx18-tdT^+^* pericytes at the transcriptional level under naïve conditions, we performed scRNA-seq analysis using cortical tissue collected from adult mice of *NG2-CreER^T2^/Ai14-flx* and *Tbx18-CreER^T2^/Ai14-flx* receiving tamoxifen treatment (100 mg/kg, i.p. for 4 consecutive days) 10 days prior to tissue collection (naïve condition) (Figure 1A). In total, we collected 10 cortices from 6 animals per genotype group (5 female cortices and 5 male cortices) for FAC sorting to collect tdT^+^/DAPI^-^ live cells for scRNA-seq (Figure 1A and Figure S1A). Moreover, we confirmed that both *NG2-CreER^T2^/Ai14-flx* and *Tbx18-CreER^T2^/Ai14-flx* have a ~70% recombination rate following tamoxifen (Figure S2A-C). We transcriptionally profiled a total of 4,722 *NG2-tdT^+^* cells and 732 *Tbx18-tdT^+^* cells (Figure 1B). After initial processing (see materials and methods), cells from both *NG2-tdT^+^* naïve group and *Tbx18-tdT^+^* naïve group were integrated for downstream analysis. Cells were clustered based on their gene expression profile and cell type annotation was based on feature gene expression and classical cell markers identified for each cluster (Figure 1C-F, Figure S1B, and Table S1). A total of 9 clusters were identified, including 5 pericyte clusters: activated pericytes cluster 0 (*Jun*^+^, *Abcc9*^+^, *Pdgfrβ*^+^, *Vtn*^+^, and *Atp13a5*^+^), canonical pericytes cluster 1 (*Abcc9*^+^, *Pdgfrβ*^+^, *Vtn*^+^, and *Atp13a5*^+^), vascular-genic pericytes cluster 3 (*Cldn5*^+^, *Ly6c1*^+^, *Abcc9*^+^, *Pdgfrβ*^+^, and *Vtn*^+^), canonical pericytes cluster 4 (*Spp1*^+^, *Atp13a5*^+^, *Abcc9*^+^, *Pdgfrβ*^+^, and *Vtn*^+^), mesenchymal-like pericytes cluster 6 (*Nnmt*^+^, *Cfh*^+^, *Rgs16*^+^, *Abcc9*^+^, *Pdgfrβ*^+^, and *Vtn*^+^). The other 4 cell clusters are composed of smooth muscle cells cluster 2 (*Acta2*^+^, *Tagln*^+^, and *Myh11*^+^), endothelial cells cluster 5 (*Cldn5*^+^, *Ly6c1*^+^, and *Klf2*^+^), OPCs cluster 7 (*Pdgfrα*^+^ and *Ptprz1*^+^) and mature oligodendrocytes (OLs) cluster 8 (*Mog*^+^, *Cnp*^+^, and *Plp1*^+^). Intriguingly, *NG2-tdT^+^* naive cells and *Tbx18-tdT^+^* naïve cells represented unique and distinct compositions of the 9 clusters identified. In this regard, *NG2-tdT^+^* cells are primarily comprised of activated pericytes (cluster 0), canonical pericytes (cluster 1), and smooth muscle cells (cluster 2), with a minimal number of canonical pericytes (cluster 4), mesenchymal-like pericytes (cluster 6), vascular-genic pericytes (cluster 3), OPCs (cluster 7), and mature OLs (cluster 8). Alternatively, *Tbx18-tdT^+^* cells are primarily composed of vascular-genic pericytes (cluster 3), activated pericytes (cluster 0), canonical pericytes (cluster 1), smooth muscle cells (cluster 2) and endothelial cells (cluster 5), with minimal expression of canonical pericytes (cluster 4), and mesenchymal-like pericytes (cluster 6, Figure 1D-E). We also noticed that the presence of non-mural cells was highly strain dependent. Unsurprisingly, only the *NG2-tdT*^+^ naïve cells contained small populations of OPCs and OLs, which were not found in the naïve *Tbx18-tdT^+^* cells (Figure 1D-E). Interestingly, endothelial cells (cluster 5) and vascular-genic pericytes (cluster 3), which are minimally present in the *NG2-tdT^+^* naïve cells, make up ~50% of total naïve *Tbx18-tdT^+^* cells (Figure 1C-E), suggesting that *Tbx18^+^* pericyte may have a specific and unique vascular-genic potential. Moreover, these strain-dependent distinctions in pericyte subpopulation proportion hold true when non-pericyte clusters are removed (Figure S1C-F) and when analyzed alongside publicly available *Tbx18-tdT^+^* data (Figure S3A-D) 33. Subsequently, pseudotime analysis using Monocle3 demonstrated that all mural cell clusters were earlier in pseudotime than the endothelial cell cluster. Moreover, the OPC cluster was earlier than the mature OL cluster (Figure 1G).

To further validate our scRNA-seq results showing that NG2 and Tbx18 mark two distinct subpopulations of pericytes, we performed immunohistochemistry for Tbx18 in *NG2-CreER^T2^/YFP-flx* mice receiving tamoxifen treatment 10 days prior and observed that only ~4% of *NG2-YFP^+^* cells expressed the Tbx18 protein and a similarly small percentage of Tbx18^+^ cells expressing NG2-YFP (Figure 1H-J). In summary, under a naïve condition (no injury), *NG2^+^* pericytes and *Tbx18^+^* pericytes represent two distinct pericyte populations.

### *NG2^+^* pericytes show strong neurogenic potential following ischemic stroke by reprogramming into radial glial cells

**Figure 2:**
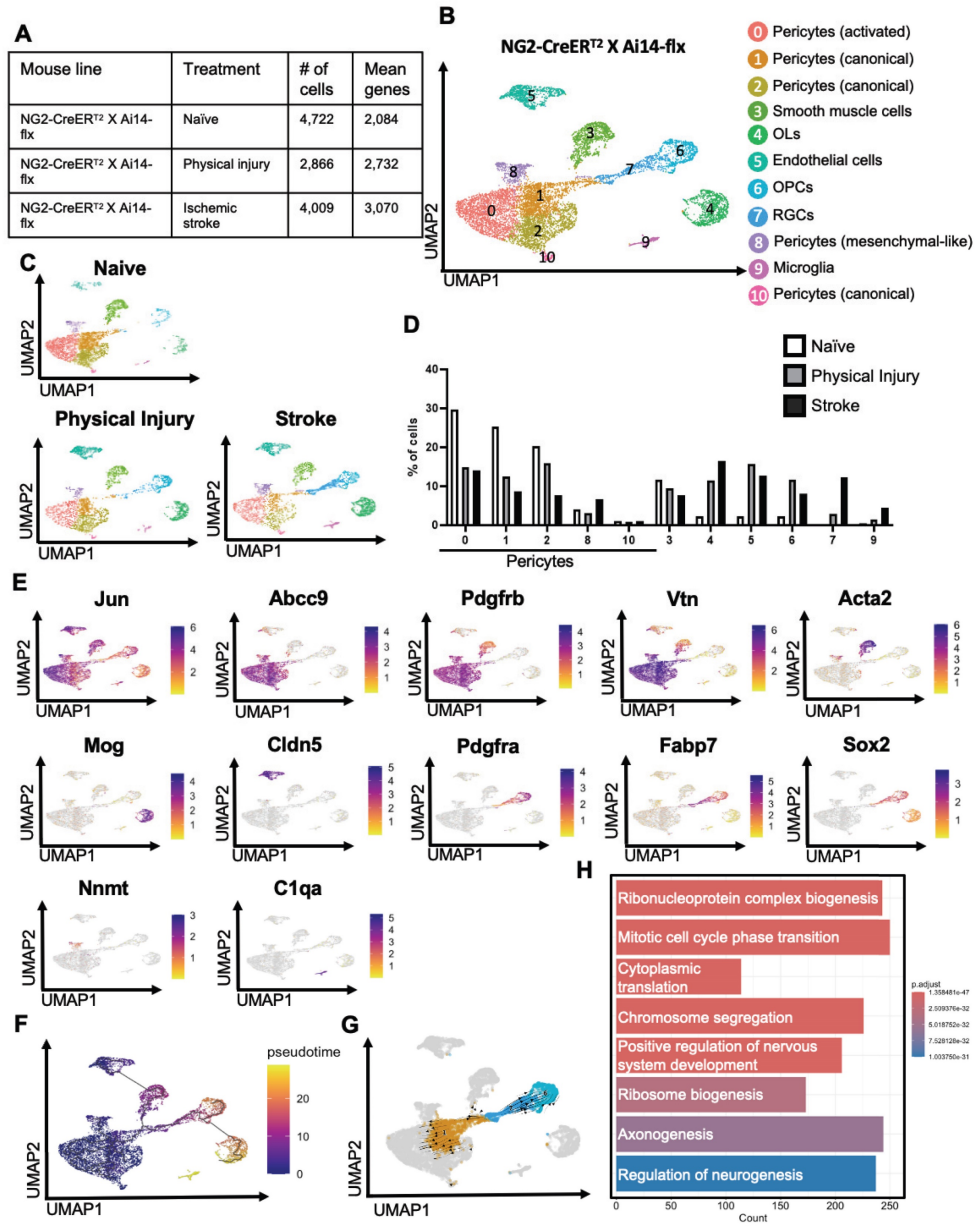
***NG2^+^* pericytes show strong neurogenic potential following ischemic stroke by reprogramming into radial glial cells.** (A) Number of cells obtained and mean number of genes per cell for naïve, physical injury, and ischemic stroke conditions obtained from *NG2-tdT^+^* mice. (B-C) Visualization of cells from *NG2-tdT^+^* naïve, physical injury, and ischemic stroke after PCA and UMAP, coloured by Seurat clustering and annotated by cell type. (D) The proportion of cells in each cluster for naïve, physical injury, and ischemic stroke obtained from *NG2-tdT^+^* mice. (E) Visualization of the total *NG2-tdT^+^* cell population after PCA and UMAP, coloured by expression of key marker genes (*Jun, Abcc9, Pdgfrβ, Vtn, Acta2, Mog, Cldn5, Pdgfrα, Fabp7, Sox2, Nnmt,* and* C1qa*). (F) Visualization of the total *NG2-tdT^+^* cell population colored by pseudotime using Monocle3. (G) Velocity vectors for the total *NG2-tdT^+^* cell population, visualized and calculated from RNA velocity using the dynamic model, projected onto UMAP visualizations of clusters 1, 6, and 7. (H) GO enrichment (biological process) results of upregulated differentially expressed genes in cluster 7 (RGPs) compared to cluster 1 (canonical pericytes). Log2 fold-change > 0.25 and p-value adjusted < 0.05.

To examine whether physical and ischemic injuries could induce multipotency of *NG2-tdT^+^* pericytes to be reprogrammed into multiple non-mural cell types, we performed scRNA-seq analysis using injured and uninjured cortical tissues collected from adult mice of *NG2-CreER^T2^/Ai14-flx* receiving tamoxifen treatment (100 mg/kg, i.p. for 4 consecutive days) 7 days prior to ET-1/L-NAME (or saline) intracerebral injections (Figure 1A). In total, we collected 10-12 cortices from 10-12 animals per group (equal number of female and male cortices) for FAC sorting to collect *tdT^+^*/DAPI^-^ live cells for scRNA-seq (Figure 1A). We transcriptionally profiled a total of 4,722 *NG2-tdT^+^* from the naïve group, 2866 *NG2-tdT^+^* cells from the physical injury group (saline injections) and 4009 *NG2-tdT^+^* cells from the ischemic injury group (ET-1/L-NAME injections) (Figure 2A). After initial pre-processing, cells from *NG2-tdT^+^* naïve, physical injury and ischemic injury groups were integrated for downstream analysis. Cells were clustered based on their gene expression profile and cell type annotation was based on feature gene expression and classical cell markers identified for each cluster (Figure 2B, E, Figure S4A, and Table S2). A total of 11 clusters of cells were identified, including 5 clusters of pericytes (0, 1, 2, 8, 10) defined as activated (cluster 0), canonical (cluster 1,2 and 10), and mesenchymal-like (cluster 8) subtypes of pericytes. The other 6 clusters of cells comprise smooth muscle cells (cluster 3), OPCs (cluster 6), mature OLs (cluster 4), endothelial cells (cluster 5), microglia (cluster 9, C1qa^+^, C1qb^+^, C1qc^+^, and Ccl4^+^), and radial glial precursors (RGPs) (34, cluster 7, Fabp7^+^, Sox2^+^, Top2a^+^, Dbi^+^, Cdca8^+^) (Figure 2B-E and Figure S4A-C). Intriguingly, the percentage of activated and canonical pericyte clusters (0, 1, 2) in total *NG2-tdT^+^* cells was drastically reduced in physical and ischemic injured groups relative to the naïve group. Simultaneously, a robust increase in the number of endothelial cells (cluster 5), OPCs (cluster 6) and OLs (cluster 4) in both physical and ischemic injured groups was observed (Figure 2D). While the smooth muscle cell population (cluster 3) remained unchanged among the three groups, the cluster of RGPs (cluster 7) and microglia (cluster 9) populations which were barely visible in the naïve group were drastically increased following ischemic injury (Figure 2D).

Based on the proximity among the RGPs (cluster 7), the canonical pericytes (cluster 1), and the OPCs (cluster 6) (Figure 2B-C), we postulate that *NG2-tdT^+^* pericytes, but not *NG2-tdT^+^* OPCs, give rise to RGPs in response to both physical and ischemic injuries. This postulation was validated using Monocle3 pseudotime analysis which showed that pericyte, RGP and OPC clusters were connected sequentially with pericytes present at the earliest time point, subsequently followed by RGPs and OPCs (Figure 2F). We then conducted trajectory inference using RNA velocity from scVelo 35 to study the full transcriptional dynamics of splicing kinetics. To do this, scVelo utilizes the relative abundance of nascent (unspliced) and mature (spliced) mRNA in each cell as an indicator of the future state of the cell. This method allowed us to describe the direction and speed with which cells transition between clusters. RNA velocity vector orientation suggests that it is *NG2-tdT^+^* pericytes (cluster 1) to give rise to RGPs (cluster 7), which subsequently make a trajectory towards OPCs (cluster 6) (Figure 2G). Finally, visualization of *NG2-tdT^+^* cells coloured by cell cycle phase revealed that RGPs (cluster 7) are primarily in the G2/M phase, whereas adjacent pericytes and OPCs are primarily in the G1 phase (Figure S4B). This suggests that RGPs are uniquely dividing or in preparation for cell division. This was further confirmed by gene ontology (GO) which demonstrated that GO (biological process) pathways “ribosome biogenesis” and “mitotic cell cycle phase transition” are enriched in cluster 7 (RGPs) compared to adjacent cluster 1 (canonical pericytes) (Figure 2H and Figure S5A-B). Moreover, further supporting the neurogenic potential of injury-induced RGPs, GO pathway “regulation of neurogenesis” was enriched in cluster 7 (RGPs) compared to adjacent cluster 1 (canonical pericytes), marked by genes such as Sox2, Olig2, Sox11, Sox10, Stmn4, Map1b, Sema3d, Bmp7, Foxg1, Ezh2, Dab1, Nkx2-2, Ascl1, Serpine2, Vim, Vgf (Figure 2H and Figure S5A-B). In addition, GO enrichment (cellular component) pathways demonstrated that genes involved in postsynaptic membrane, density and specialization and ribosome subunits were enriched in cluster 7 (RGPs) relative to adjacent canonical pericyte cluster 1, arguing dominant neurogenic potential of RGPs following injury (Figure S5B).

**Figure 3:**
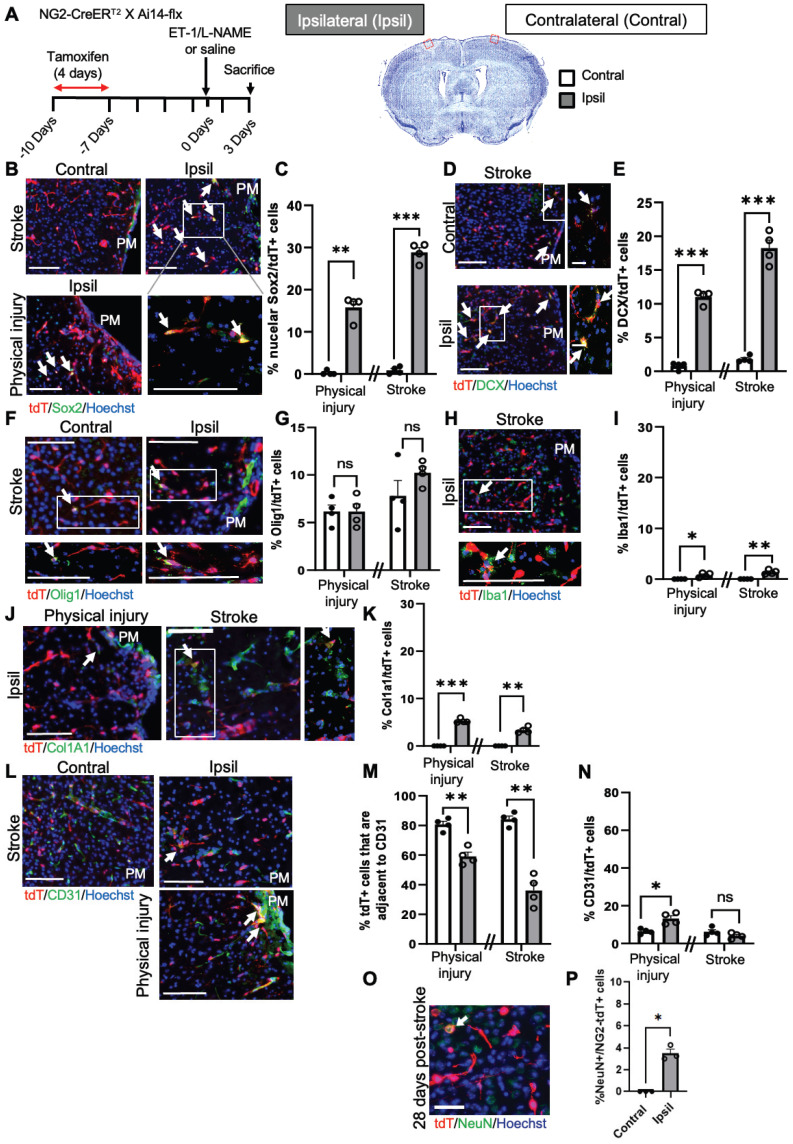
***NG2^+^* pericytes exhibit strong neural reprogramming potential following brain injury.** (A) Flowchart of brain injury induced by intracerebral injections of ET-1/L-NAME (or saline) into the sensory-motor cortex of *NG2-CreER^T2^*/*Ai14-flx* mice receiving tamoxifen treatment 7 days prior to injury and sacrificed at 3 days after injury for immunohistochemistry. Cresyl violet image of a brain section at 3 days post-stroke. The red box shows where representative immunohistochemical images were taken. (B-C) Images and quantitative analysis of the proportion of Sox2^+^/*tdT^+^* i-NSCs in the cortex sections from mice receiving ET-1/L-NAME (stroke) or saline (physical injury) injections, immunostained for Sox2 (green) and tdT (red), and counterstained for Hoechst (blue). Scale bar: 100 µm. (D-E) Images and quantitative analysis of the percentage of DCX^+^/*tdT^+^* neuroblasts in the cortex sections, immunostained for DCX (green) and tdT (red) and counterstained for Hoechst (blue). Scale bar: 100 µm (left panel); 50 µm (right panel). White boxes in the left panels were enlarged in the right panels. (F-G) Images and quantitative analysis of the percentage of Olig1^+^/*tdT^+^* OL lineage cells in the cortex sections, immunostained for Olig1 (green) and tdT (red) and counterstained for Hoechst (blue). Scale bar: 100 µm. (H-I) Images and quantitative analysis of the proportion of Iba1^+^/*tdT^+^* microglia in the cortex sections, immunostained for Iba1 (green) and tdT (red) and counterstained for Hoechst (blue). Scale bar: 100 µm. White boxes in the upper panels were enlarged in the bottom panels. (J-K) Images and quantitative analysis of the percentage of Col1a1^+^/*tdT^+^* fibroblasts in the cortex sections, immunostained for Col1a1 (green) and tdT (red) and counterstained for Hoechst (blue). Scale bar: 100 µm (left panel); 50 µm (right panel). White boxes in the left panels were enlarged in the right panels. (L-M) Images and quantitative analysis of the proportion of NG2-tdT^+^ cells that were adjacent to CD31^+^ micro-vessels, immunostained for CD31 (green) and *tdT^+^* (red) and counterstained for Hoechst (blue). Scale bar: 100 µm. (N) Quantitative analysis of the percentage of CD31^+^/*tdT^+^* micro-vessels, as shown in (L) in the cortex sections. Arrows denote co-labelled cells. PM: Pia Mater. (O-P) Images and quantitative analysis of the proportion of NeuN^+^/tdT^+^ in the cortex 28 days post-stroke, immunostained for NeuN (green) and tdT (red), and counterstained for Hoechst (blue). Scale bar: 50 µm. n=3-4 animals/group. Student t-test, *P < 0.05; **P < 0.01; ***P < 0.001.

To further confirm the above scRNA-seq analysis, we performed immunohistochemistry 1 and 3 days after ET-1/L-NAME (or saline) injections with *NG2-CreER^T2^/Ai14-flx* mice receiving tamoxifen treatment (100 mg/kg, i.p. daily for 4 days) 7 days prior to injury (Figure 3A). We found that 28% of NG2-tdT*^+^* cells were Sox2^+^ i-NSCs at the site of stroke injury at 3 days post-injury (dpi), while only 10% of NG2-tdT^+^ cells were Sox2^+^ i-NSCs at 1 dpi (Figure 3B-C and Figure S6A-B). No NG2-tdT^+^/Sox2^+^ cells were observed at the contralateral side of the uninjured cortex (Figure 3B-C). On the other hand, physical injury caused by saline injections induced 15% of NG2-tdT^+^ cells expressing Sox2 (Figure 3B-C) at the site of injury. We further immunostained for the neuroblast marker, doublecortin (DCX), and observed that 18% of NG2-tdT^+^ cells at the site of stroke injury and 11% of NG2-tdT*^+^* cells at the site of physical injury were positive for DCX, while less than 1% NG2-tdT*^+^*/DCX^+^ cells were observed at the contralateral side of uninjured cortices (Figure 3D-E). No NG2-tdT*^+^*/ DCX^+^ cells were observed at 1-day post-stroke. Intriguingly, the population of *NG2-tdT^+^*/Olig1^+^ OLs was not altered following both physical and ischemic stroke injuries, at the range of between 6% and 10% of NG2-tdT^+^ cells (Figure 3F-G). As shown in scRNA-seq analysis (Figure 2D), ischemic stroke injury induced higher numbers of Iba1^+^ microglia derived from NG2-tdT*^+^* than physical injury did (Figure 3H-I and Figure S6C), and physical injury triggered higher numbers of Col1a1^+^ fibroblasts from NG2-tdT^+^ than ischemic stroke injury did (Figure 3J-K and Figure S6D). Consistent with the neural and non-neural reprogramming capabilities of pericytes following injury (Figure 2), we showed that the percentage of NG2-tdT*^+^* cells that were adjacent to CD31^+^ micro-vessels were significantly reduced following both physical and ischemic stroke injuries (Figure 3L-M). Intriguingly, physical injury significantly induced the genesis of CD31^+^ micro-vessels from NG2-tdT^+^ cells but ischemic stroke injury did not (Figure 3L, N and Figure S6E). We further performed a long-term lineage tracing experiment using the *NG2-CreER^T2^/Ai14-flx* mouse line (Figure 3O-P). Immunohistochemical analysis of brain sections from 28 days post-stroke injury showed that there were 3% of NG2-tdT*^+^* cells expressing NeuN, a marker for mature neurons in the injured cortical tissues, while no NeuN^+^/NG2-tdT^+^ co-labelled cells were observed in the contralateral cortices. This result was consistent with a recently published paper using the *NG2-CreER^T2^/Ai14-flx* mouse line for long-term lineage tracing experiments in the middle cerebral artery occlusion (MCAO) stroke model 26. In summary, NG2*^+^* pericytes exhibit robust neural reprogramming potential following injury, particularly after ischemic stroke injury, while showing modest potential to produce microglia, fibroblasts and micro-vessels following injury.

### *Tbx18^+^* pericytes exhibit strong vascular-genic potential following ischemic stroke

**Figure 4:**
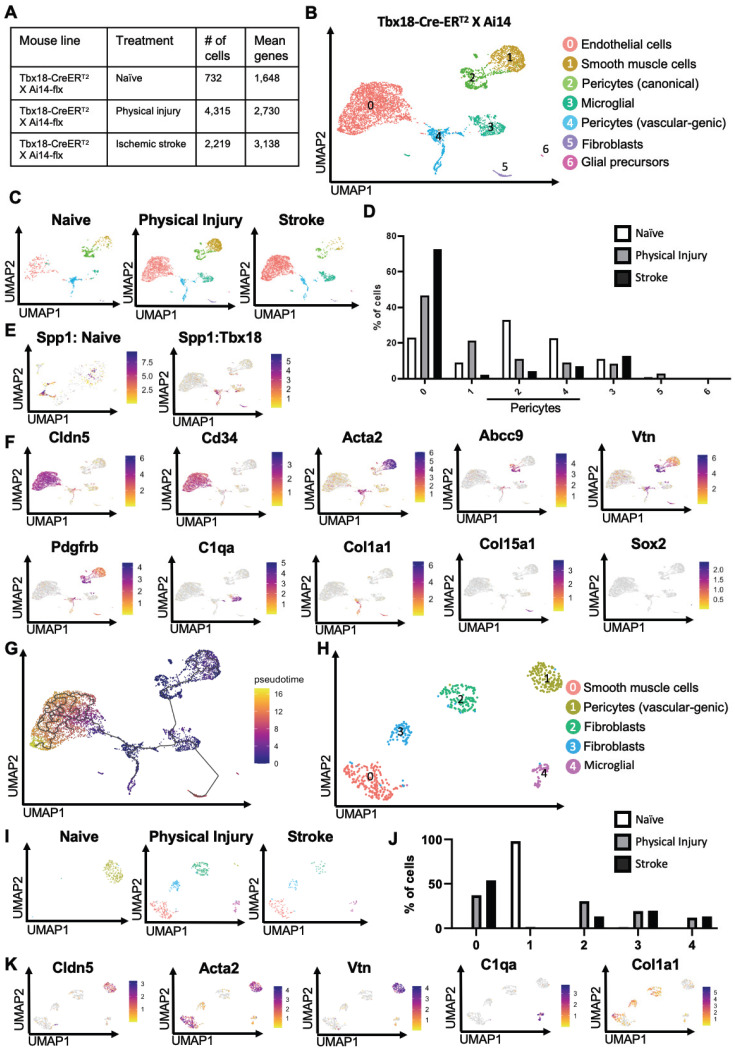
***Tbx18^+^* pericytes exhibit strong vascular-genic potential following ischemic stroke.** (A) Number of cells obtained and mean number of genes per cell for naïve, physical injury, and ischemic stroke conditions obtained from *Tbx18-tdT^+^* mice. (B-C) Visualization of cells from *Tbx18-tdT^+^* naïve, physical injury, and ischemic stroke after PCA and UMAP, coloured by Seurat clustering and annotated by cell type. (D) The proportion of cells in each cluster for naïve, physical injury, and ischemic injury was obtained from *Tbx18-tdT^+^* mice. (E) *Spp1* expression visualized in total *Tbx18-tdT^+^* population and in integrated naïve *NG2-tdT^+^* and naïve *Tbx18-tdT^+^* population after PCA and UMAP. (F) Visualization of the total *Tbx18-tdT^+^* cell population after PCA and UMAP, coloured by expression of key marker genes (*Cldn5, Cd34, Acta2, Abcc9, Vtn, Pdgfr*β*, C1qa, Col1a1, Col15a1,* and* Sox2*). (G) Visualization of the total *Tbx18-tdT^+^* cell population, coloured by pseudotime using Monocle3. (H-I) Visualization of cells subset from *Tbx18-tdT^+^* vascular-genic pericytes in naïve, physical injury, and ischemic stroke after PCA and UMAP, coloured by Seurat clustering and annotated by cell type. (J) The proportion of cells in each subcluster for cells subset from* Tbx18-tdT^+^* vascular-genic pericytes in naïve, physical injury, and ischemic stroke. (K) Visualization of the cell subsets from *Tbx18-tdT^+^* vascular-genic pericytes after PCA and UMAP, coloured by expression of key marker genes (*Cldn5, Acta2, Vtn, C1qa,* and *Col1a1*).

To examine whether physical and ischemic injuries could induce multipotency of *Tbx18-tdT^+^* pericytes to be reprogrammed into multiple non-mural cell types, we performed scRNA-seq analysis using injured and uninjured cortical tissues collected from adult mice of *Tbx18-CreER^T2^/Ai14-flx* receiving tamoxifen treatment (100 mg/kg, i.p. for 4 consecutive days) 7 days prior to ET-1/L-NAME (or saline) intracerebral injections (Figure 1A). In total, we collected 10-12 cortices from 10-12 animals per group (equal number of female and male cortices) for FAC sorting to collect *tdT^+^/*DAPI^-^ alive cells for scRNA-seq (Figure 1A). We transcriptionally profiled a total of 732 *Tbx18-tdT^+^* cells from the naïve group, 4315 *Tbx18-tdT^+^* cells from the physical injury group (saline injections) and 2219 *Tbx18-tdT^+^* cells from the ischemic stroke group (ET-1/L-NAME injections) (Figure 4A). After pre-processing, the three *Tbx18^+^* groups including naïve, physical injury, and ischemic stroke, were integrated for downstream analysis (Figure 4B-F, Figure S7A, and Table S2). The analysis revealed seven different cell types including endothelial cells (cluster 0, Cldn5^+^, *Flt1*^+^, *Ly6c1*^+^, *Cd34*^+^), smooth muscle cells (Cluster 1, *Acta2*^+^, *Myh11*^+^, *Tagln*^+^), canonical pericytes (cluster 2, *Vtn*^+^, *Abcc9*^+^, *Atp13a5*^+^, *Rgs5*^+^), microglia (cluster 3, *Cd74*^+^, *C1qa*^+^), vascular-genic pericytes (cluster 4, *Pdgfrβ*^+^, *Spp1*^+^, *Vtn*^+^, *Acta2*^+^, Cldn5^+^), fibroblasts (cluster 5, *Col1a1*^+^, *Col15a1*^+^, *Dcn*^+^) and glial precursors (cluster 6, Pcdh1^+^. Slc1a2^+^, Sox2^+^). Important to note, that cluster 6 glial precursors, are negative for key RGP proliferation makers, such as *Mki67* (34, Figure S7B). Intriguingly, the vascular-genic pericyte cluster only present in *Tbx18-tdT^+^* lineage groups (Figure 1C-E, and Figure 4B-D) expressed *Spp1*, a gene found in a subset of pericytes (36, Figure 4E), and* Cldn5* (Figure 4F). This suggests that a subpopulation of Tbx18 pericytes have a distinct ability to produce a vascular linage. Under both physical and ischemic injury conditions, the vascular-genic pericyte (cluster 4) and canonical pericyte populations (cluster 2) were reduced, while simultaneously, the number of endothelial cells (cluster 0) was robustly increased (Figure 4D). This further illustrates the specific reprogramming ability of *Tbx18^+^* pericytes to produce an endothelial cell lineage.

Intriguingly, the number of smooth muscle cells (cluster 1) was drastically increased in *Tbx18-tdT^+^* cells from a physical injury group but robustly reduced from an ischemic injury group (Figure 4C-D). On the other hand, the number of cells from the fibroblast cluster increased only following physical injury but not ischemic injury (Figure 4C-D). Finally, we found that glial precursors were not present in the naïve group, but minimally produced following both physical and ischemic injuries from *Tbx18-tdT^+^* pericytes. To further confirm trajectory changes among different cell clusters in the *Tbx18-tdT^+^* cells, we performed Monocle 3 pseudotime analysis and demonstrated that endothelial cells, fibroblasts, and glial precursors were later in pseudotime relative to other mural cell populations (Figure 4G), implying vascular, fibrotic and neural potency of *Tbx18-tdT^+^* pericytes following physical and ischemic injuries with vascular-genic potency as a dominant reprogrammed cell lineage. This statement was further supported by cell atlas analysis of the subset of vascular-genic pericytes. We obtained 5 subclusters: vascular-genic pericytes, smooth muscle cells, microglia and fibroblasts (Figure 4H-J). Intriguingly, the naïve group was primarily composed of vascular-genic pericytes without other subclusters, while the vascular-genic pericyte subcluster was absent under both physical and ischemic injury conditions, where the pericyte subcluster was replaced by smooth muscle cells, microglia and fibroblasts (Figure 4J-K). This cell atlas of vascular-genic pericytes aligns with the potential for *Tbx18-tdT^+^* pericytes to gain multipotent reprogramming capabilities following physical and ischemic injuries to produce endothelial cells, fibroblasts, and microglia. In addition, we performed differentially expressed gene analysis and GO analysis of cluster 0 (endothelial cells) compared to adjacent cluster 4 (vascular-genic pericytes) (Figure S8A-B) and showed that significantly upregulated genes are involved in epithelial cell migration, tissue migration and small GTPase-mediated signal transduction, which are essential for vascular genesis and angiogenesis (Figure S8B).

**Figure 5:**
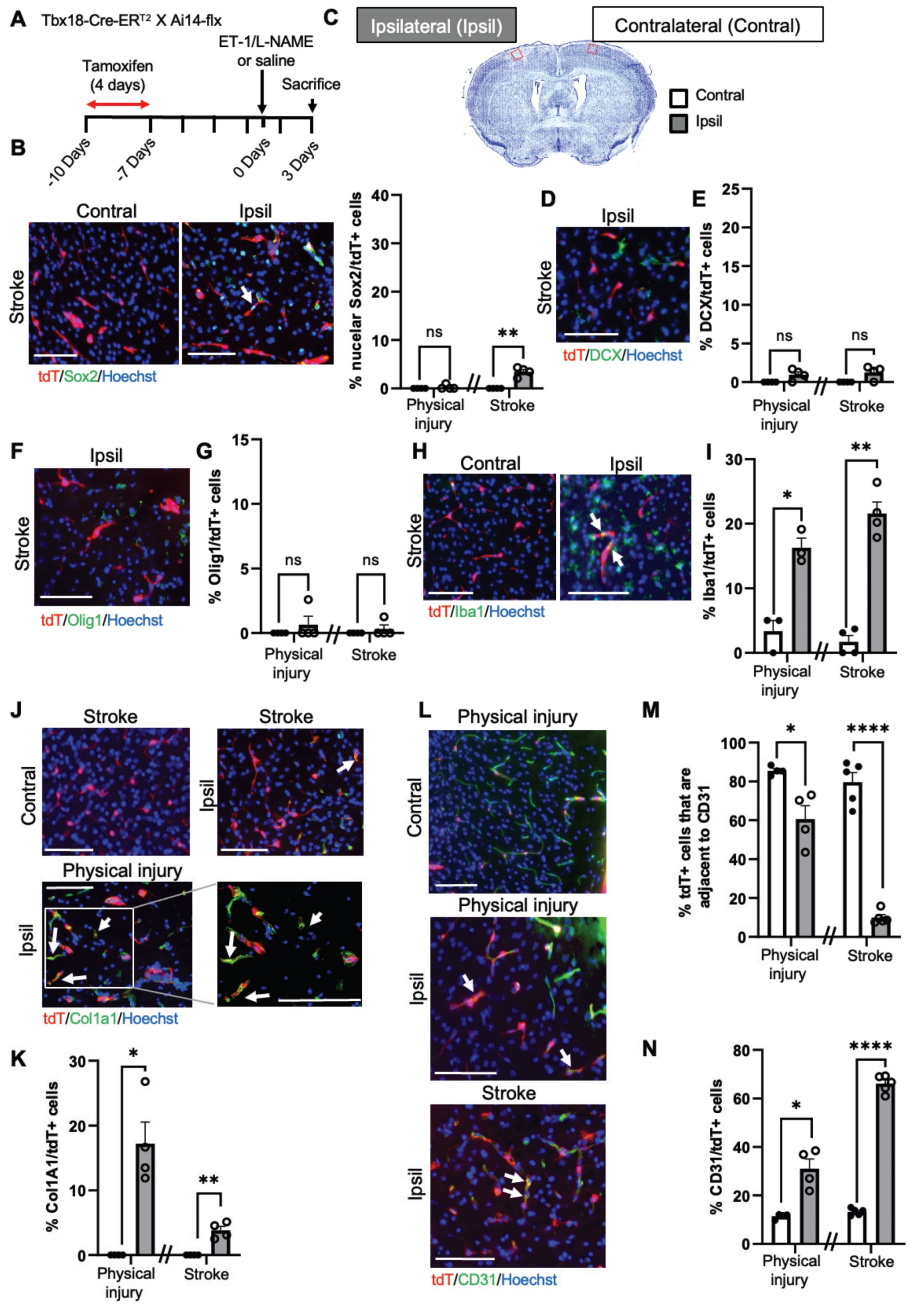
***Tbx18^+^* pericytes predominantly produce micro-vessels following brain injury.** (A) Flowchart of brain injury induced by intracerebral injections of ET-1/L-NAME (or saline) into the sensory-motor cortex of *Tbx18-CreER^T2^/Ai14-flx* mice receiving tamoxifen treatment 7 days prior to injury and sacrificed at 3 days after injury for immunohistochemistry. Cresyl violet image of a brain section at 3 days post-stroke. The red box shows where representative immunohistochemical images were taken. (B-C) Images and quantitative analysis of the proportion of Sox2^+^/tdT^+^ i-NSCs in the cortex sections from mice receiving ET-1/L-NAME (stroke) or saline (physical injury) injections, immunostained for Sox2 (green) and tdT^+^ (red) and counterstained for Hoechst (blue). (D-E) Images and quantitative analysis of the percentage of DCX^+^/tdT^+^ neuroblasts in the cortex sections, immunostained for DCX (green) and tdT^+^ (red) and counterstained for Hoechst (blue). (F-G) Images and quantitative analysis of the percentage of Olig1^+^/tdT^+^ OL lineage cells in the cortex sections, immunostained for Olig1 (green) and tdT (red) and counterstained for Hoechst (blue). (H-I) Images and quantitative analysis of the proportion of Iba1^+^/tdT^+^ microglia in the cortex sections, immunostained for Iba1 (green) and tdT^+^ (red) and counterstained for Hoechst (blue). (J-K) Images and quantitative analysis of the percentage of Col1a1^+^/tdT^+^ fibroblasts in the cortex sections, immunostained for Col1a1 (green) and tdT^+^ (red) and counterstained for Hoechst (blue). (L-M) Images and quantitative analysis of the proportion of Tbx18-tdT^+^ cells that were adjacent to CD31^+^ micro-vessels, immunostained for CD31 (green) and tdT^+^ (red) and counterstained for Hoechst (blue). (N) Quantitative analysis of the percentage of CD31^+^/tdT^+^ micro-vessels, as shown in (L) in the cortex sections. Arrows denote co-labelled cells. Scale bar: 100 µm. n=4 animals/group. Student t-test, *P < 0.05; **P < 0.01; ***P < 0.001.

To further confirm the above scRNA-seq analysis, we performed immunohistochemistry 1 and 3 days after ET-1/L-NAME (or saline) injections with *Tbx18-CreER^T2^/Ai14-flx* mice receiving tamoxifen treatment (100 mg/kg, i.p. daily for 4 days) 7 days prior to injury (Figure 5A). Unlike NG2-tdT^+^cells, Tbx18-tdT*^+^*cells exhibited minimal potential to produce neural lineage cells following injury (Figure 5B-G). Although Tbx18-tdT*^+^* cells can generate a small number of Sox2^+^ i-NSCs following ischemic stroke at both 1 and 3 dpi (Figure 5B-C, and Figure S9A-B), they barely produce DCX^+^ neuroblasts and Olig1^+^ OL lineage cells (Figure 5D-G and Figure S9D-E). On the other hand, we observed that both physical injury and ischemic injury can reprogram Tbx18-tdT*^+^* pericytes into Iba1^+^ microglia and Col1a1^+^ fibroblasts at the range of 10%-20% (Figure 5H-K). As expected, the percentage of Tbx18-tdT*^+^* cells that are adjacent to CD31^+^ micro-vessels were significantly reduced following both physical injury and ischemic stroke injury (Figure 5L-M). Consistent with scRNA-seq analysis (Figure 4D), there was a robust population of CD31^+^ micro-vessels derived from Tbx18-tdT^+^ cells following ischemic injury (Figure 5L, N). Together, Tbx18^+^ pericytes exhibit robust non-neural reprogramming potential following injury, particularly generating new micro-vessels, while exhibiting minimal potential to produce neural lineage cells following injury.

### CBP Ser436 dephosphorylation/phosphorylation modulates pericyte reprogramming/differentiation process by regulating acetylation shift between Sox2 and H2B and Sox2 nuclear-cytoplasmic trafficking

**Figure 6:**
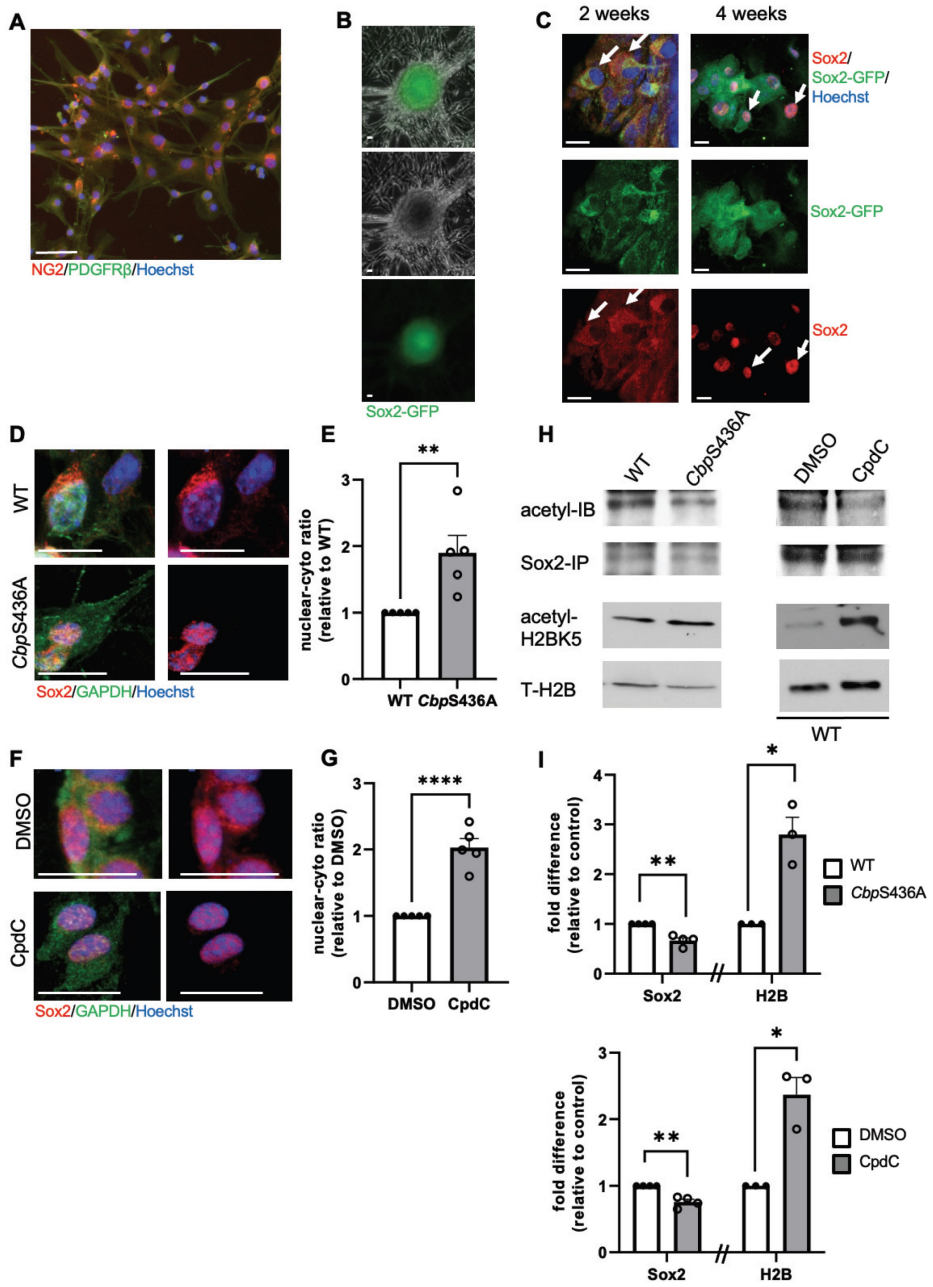
** Inactivation of the aPKC-CBP pathway induces an acetylation shift from Sox2 to H2B and Sox2 nuclear import during i-NSC reprogramming from a-pericytes.** (A) a-pericytes expanded on an uncoated plastic dish were immunostained with pericyte markers, NG2 (red) and Pdgfrβ (green), counterstained with Hoechst (blue). (B-C) i-neurospheres were produced from cultured a-pericytes isolated from stroke-injured Sox2-GFP reporter mouse cortical tissues when treated with neural conditioned medium (NCM) and expressed GFP signal (B); cytospin of these i-neurospheres and immunocytochemistry for Sox2 (red), GFP (green), counterstained for Hoechst (blue) at 2 and 4 weeks upon reprogramming (NCM treatment, C). (D-G) Confocal images and quantitative analyses of Sox2 nucleus/cytosol intensity ratio in WT and *Cbp*S436A i-NSCs (D-E), in the absence and presence of CpdC (F-G) at 3 weeks upon reprogramming. (H) Immunoprecipitation analysis of Sox2 acetylation in WT and *Cbp*S436A i-NSCs (left panels) or in the absence and presence of CpdC (right panels). i-NSC lysates were immunoprecipitated with a Sox2 antibody, washed and then blotted with pan-acetyl and Sox2 antibodies. Western blot analysis for H2BK5 acetylation in i-NSCs from WT and *Cbp*S436A mice. Blots were probed for acetyl-H2BK5 and total H2B. (I) Graphs show relative levels of acetylation of Sox2 and H2BK5 over total Sox2 and H2B, respectively, normalized to control samples (WT and WT-DMSO, respectively). Scale bar: 20 µm. ***P < 0.05; n=3-5 animals/group.

Previously, we cultured NG2^+^/Pdgfrβ^+^ a-pericytes isolated from ischemic injured cortices and were able to reprogram them into multipotent neural stem cells (NSCs) by forming induced-neurospheres (i-neurospheres) upon exposure to neural conditioned medium (NCM) (16). Here, we performed the same reprogramming culture experiment using a Sox2-GFP reporter mouse line and showed that i-neurospheres expressed GFP, while the adjacent monolayer cells in the plate were negative for GFP (Figure 6A-B). When we further performed cytospin of these GFP^+^ i-neurospheres, we observed that Sox2 protein initially accumulated in the cytoplasm of the cells 2 weeks upon NCM treatment and that it was transported back into the nucleus 4 weeks after NCM treatment to complete reprogramming (Figure 6C).

Since we previously showed that both *CbpS436A* knock-in mice and CpdC treatment can facilitate neural reprogramming efficiency of a-pericytes (16), here we demonstrated that *CbpS436A* a-pericytes and CpdC treatment (5 µM) during the neural reprogramming process significantly accelerated Sox2 nuclear import, measured by an increased nuclear/cytoplasmic ratio of Sox2 intensity in *CbpS436A* i-NSCs (Figure 6D-E) and in WT i-NSCs treated with CpdC (Figure 6F-G). To further assess whether this increased Sox2 nuclear import was associated with an acetylation shift between Sox2 and H2B, we performed western blot analysis to measure H2BK5 acetylation, a dominant lysine mark acetylated by CBP 37-39. In addition, we conducted immunoprecipitation with an anti-Sox2 antibody to measure acetylation of Sox2 using a pan acetyl-antibody. Interestingly, we observed that both *CbpS436A* i-NSCs and WT CpdC-treated i-NSCs exhibited increased H2BK5 acetylation but reduced Sox2 acetylation (Figure 6H-I). Thus, inactivation of the aPKC-CBP pathway shifted acetylation away from Sox2 towards H2B and accelerated Sox2 nuclear import to promote the reprogramming of a-pericytes into i-NSCs, as described in our previous study 16.

**Figure 7:**
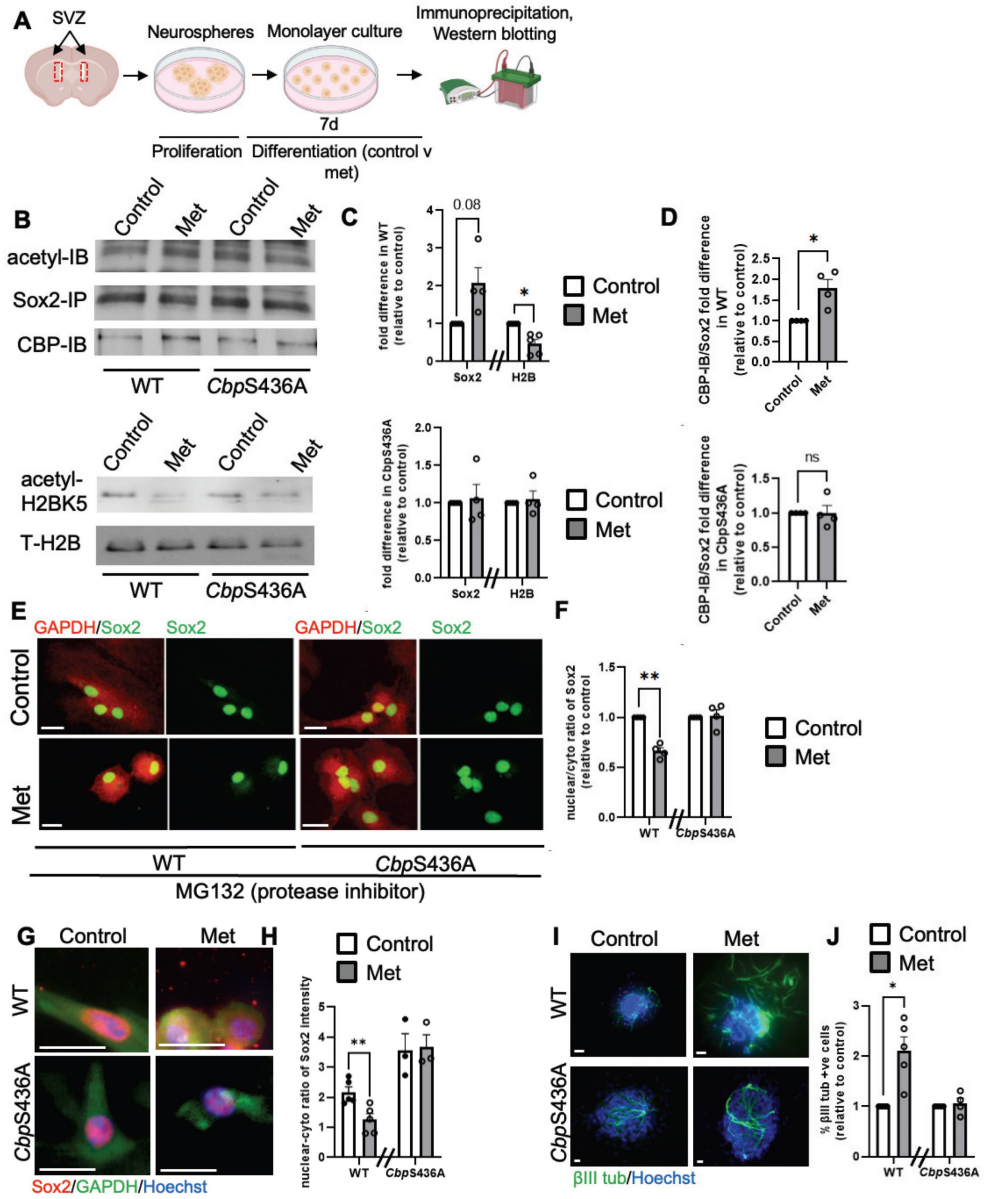
** Activation of the aPKC-CBP pathway induces an acetylation shift from H2B to Sox2 and Sox2 nuclear export during neuronal differentiation of SVZ NPC and i-NSC.** (A) Schematic of experimental design for the differentiation of SVZ NPCs using a neurosphere culture model. (B) Immunoprecipitation analysis of Sox2 acetylation in WT and *Cbp*S436A differentiating SVZ NPCs in the absence and presence of metformin (left panels). SVZ NPCs lysates were immunoprecipitated with a Sox2 antibody, washed and then blotted with pan-acetylated, CBP and Sox2 antibodies. Western blot analysis for H2BK5 acetylation in SVZ NPCs from WT and *CbpS436A* mice (right panels). Blots were probed for acetyl-H2BK5 and total H2B. (C) Graphs show relative levels of acetylation of Sox2 and H2B over total Sox2 and H2B, respectively, normalized to controls for WT (top panel) and *CbpS436A* (bottom panel) SVZ NPCs without metformin. (D) Graphs show relative levels of CBP-IB over total pulled-down Sox2, normalized to controls for WT (top panel) and *Cbp*S436A (bottom panel) SVZ NPCs in the absence of metformin. (E-F) Confocal images and quantitative analyses of Sox2 nucleus/cytosol intensity ratio in WT and *CbpS436A* SVZ NPCs 6 days upon neuronal differentiation in the absence and presence of metformin and treated with MG132 (1 µM) 16 h prior to fixation. (G-H) Confocal images and quantitative analyses of Sox2 nucleus/cytosol intensity ratio in WT and *CbpS436A* i-NSCs 2 days upon neuronal differentiation in the absence and presence of metformin. (I-J) Photographs and quantitative analyses of the percentage of βIII tubulin-positive newly born neurons from WT and *CbpS436A* i-NSCs 7 days upon neuronal differentiation in the absence and presence of metformin. Scale bar: 20 µm. **P < 0.01; *P < 0.05; n=3-5 animals/group.

In contrast, our previous work demonstrated that metformin, an AMPK activator, promoted neuronal differentiation of adult subventricular zone (SVZ) NPCs by activating the aPKC-CBP pathway to fully phosphorylate CBP at Ser436 27. To ask whether the increased neuronal differentiation by metformin is associated with Sox2 nuclear export driven by increased Sox2 acetylation at the expense of H2B acetylation, we first examined H2BK5 acetylation and Sox2 acetylation in differentiating SVZ NPCs derived from WT and *CbpS436A* mice in the absence and presence of metformin (1 µM). We showed that metformin enhanced Sox2 acetylation while reducing H2BK5 acetylation in WT SVZ NPCs, but it did not alter Sox2 and H2B acetylation in *CbpS436A* SVZ NPCs (Figure 7A-D). Since both Sox2 and H2B have been shown as important substrates of CBP, we further asked whether metformin-induced CBP S436 phosphorylation favored CBP binding to Sox2 for enhancing Sox2 acetylation. In this regard, we performed co-immunoprecipitation (co-IP) experiments by pulling down Sox2 protein to assess CBP binding capability using the SVZ NPC differentiation culture model. Interestingly, metformin promoted the interaction between CBP and Sox2 in wild-type SVZ NPCs, while metformin lost the ability in *CbpS436A* SVZ NPCs (Figure 7A-B, D). These data support that CBPS436 phosphorylation is required for metformin to enhance the CBP binding to Sox2 that is necessary for Sox2 acetylation. Subsequently, we examined endogenous Sox2 nuclear-cytoplasmic trafficking in the differentiating SVZ NPCs in the presence of MG132, an inhibitor of proteasomal degradation to prevent Sox2 protein degradation in the cytoplasm. We observed that metformin triggered Sox2 nuclear export in the WT SVZ NPCs but not *CbpS436A* SVZ NPCs (Figure 7E-F), measured by a nuclear/cytoplasmic ratio of endogenous Sox2 protein intensity, 6 days upon differentiation and treated with MG132 (1 µM) for 16h before fixation. We additionally confirmed this effect by transfecting exogenous GFP-fused human Sox2 (hSox2) in the SVZ NPCs. The quantitative analysis showed that the percentage of differentiating NPCs that exhibited a GFP signal in both nuclear and cytoplasmic compartments was significantly increased in metformin-treated WT SVZ NPCs but not in metformin-treated *CbpS436A* SVZ NPCs 3 days upon differentiation and treated with MG132 (1 µM) for 16 h before fixation (Figure S10A-B).

Finally, we used i-NSCs derived from a-pericytes to perform a differentiation assay. Consistent with the results from SVZ NPCs, metformin was able to promote neuronal differentiation of WT i-NSCs and enhance Sox2 nuclear export, but it did not alter neuronal differentiation and Sox2 nuclear-cytoplasmic trafficking in *CbpS436A* i-NSCs (Figure 7G-J). Together, metformin treatment activates the aPKC-CBP pathway to shift acetylation away from H2B towards Sox2, thus facilitating Sox2 nuclear export upon differentiation. In summary, inactivation of AMPK-stimulated CBPS436 phosphorylation can facilitate neural reprogramming of a-pericytes by enhancing Sox2 nuclear import and shifting acetylation away from Sox2 towards H2B, while activation of AMPK-stimulated CBPS436 phosphorylation can enhance neuronal differentiation of i-NSCs derived from a-pericytes via increased Sox2 nuclear export and acetylation shift towards Sox2 (Figure 7G-H and Figure S10C).

### Sequential treatment of CpdC and metformin facilitates reprogramming /differentiation of *NG2^+^* pericytes into functional neurons in culture

After identifying local i-NSCs derived from *NG2^+^* cortical pericytes following ischemic stroke injury, we decided to model ischemic stroke *in vitro* to develop a genomic integration-free methodology to reprogram pericytes into functional neurons in culture. First, we used naïve B6129SF2/J adult mice (2-4 months old) to isolate cortical tissues for primary cortical pericyte culture as established previously (Figure S11A) under pericyte media (PM) containing 2% fetal bovine serum (FBS) and epidermal growth factor (EGF). Upon receiving hypoxic treatment (3% O2) for 5 days in pericyte conditioned media (PCM) followed by normoxic condition for 4 days in neural conditioned media (NCM) containing EGF, FGF2, leukemia inhibitory factor (LIF), and N2 (Figure S11A), the cultured pericytes were fixed at the middle stage to perform immunocytochemistry for Sox2, a marker for NSCs. Intriguingly, Sox2 was barely expressed in control pericytes, but appeared at the middle stage in cultured pericytes following hypoxic treatment in the absence and presence of CpdC (Figure S11B-C), indicating Sox2^+^ cells as pericyte-derived i-NSCs. Importantly, we characterized the Sox2^+^ cells as either nuclear or nuclear/cytoplasmic expression (Figure S11B) and showed that CpdC (5 µM) treatment robustly increased the percentage of nuclear Sox2^+^ cells at the middle stage, suggesting that CpdC could facilitate Sox2 nuclear import following hypoxia. To further differentiate these Sox2^+^ i-NSCs into newborn neurons, we continued to culture the middle-stage cells by replacing NCM with neuronal differentiation media (NDM) for 7 days after a 2-day break time in NCM to wash out CpdC (Figure S11A). Interestingly, the percentage of nuclear Sox2^+^ cells at all three differentiation conditions was actually lower than that in the middle-stage group receiving CpdC treatment, while the proportion of total Sox2^+^ Cells remained the same throughout the middle and differentiation stages (Figure S11C-D). These results suggest that Sox2 nuclear-cytoplasmic trafficking is involved in hypoxia-induced pericyte neural reprogramming and differentiation process: CpdC facilitates Sox2 nuclear import during pericyte to i-NSC reprogramming (middle stage) and Sox2 nuclear export is triggered by neuronal differentiation of i-NSCs (differentiation stage). To further examine the newborn neuron production from the hypoxia-induced pericyte reprogramming/differentiation culture, we analyzed the percentage of βIII tubulin^+^ neurons from all groups at the different stages. Intriguingly, the number of βIII tubulin^+^ neurons was significantly higher at the differentiation stages relative to the control group and middle-stage group. Of all the groups assessed in the differentiation stage (Figure S11E-F), the group receiving CpdC at the middle stage followed by metformin at the differentiation stage exhibited the highest production of βIII tubulin^+^ neurons.

Next, we cultured isolated cortical pericytes from both *NG2-CreER^T2^/Ai14-flx* and *Tbx18-CreER^T2^/Ai14-flx* mice and used 4-hydroxytamoxifen (4-OH-TAM, 1 µM) to induce recombination in both lines before hypoxia treatment (Figure S12A-B). Interestingly, we found that Tbx18-tdT^+^ cells barely produced βIII tubulin^+^ neurons and the percentage of βIII tubulin^+^/NG2-tdT*^+^* was significantly greater than βIII tubulin^+^/Tbx18-tdT^+^ (Figure S12A-B). These results suggest that NG2-tdT^+^ pericytes have much stronger neural reprogramming/differentiation potency than Tbx18-tdT pericytes following hypoxia in culture, reminiscence of NG2-tdT^+^ pericytes neural reprogramming feature *in vivo*.

**Figure 8:**
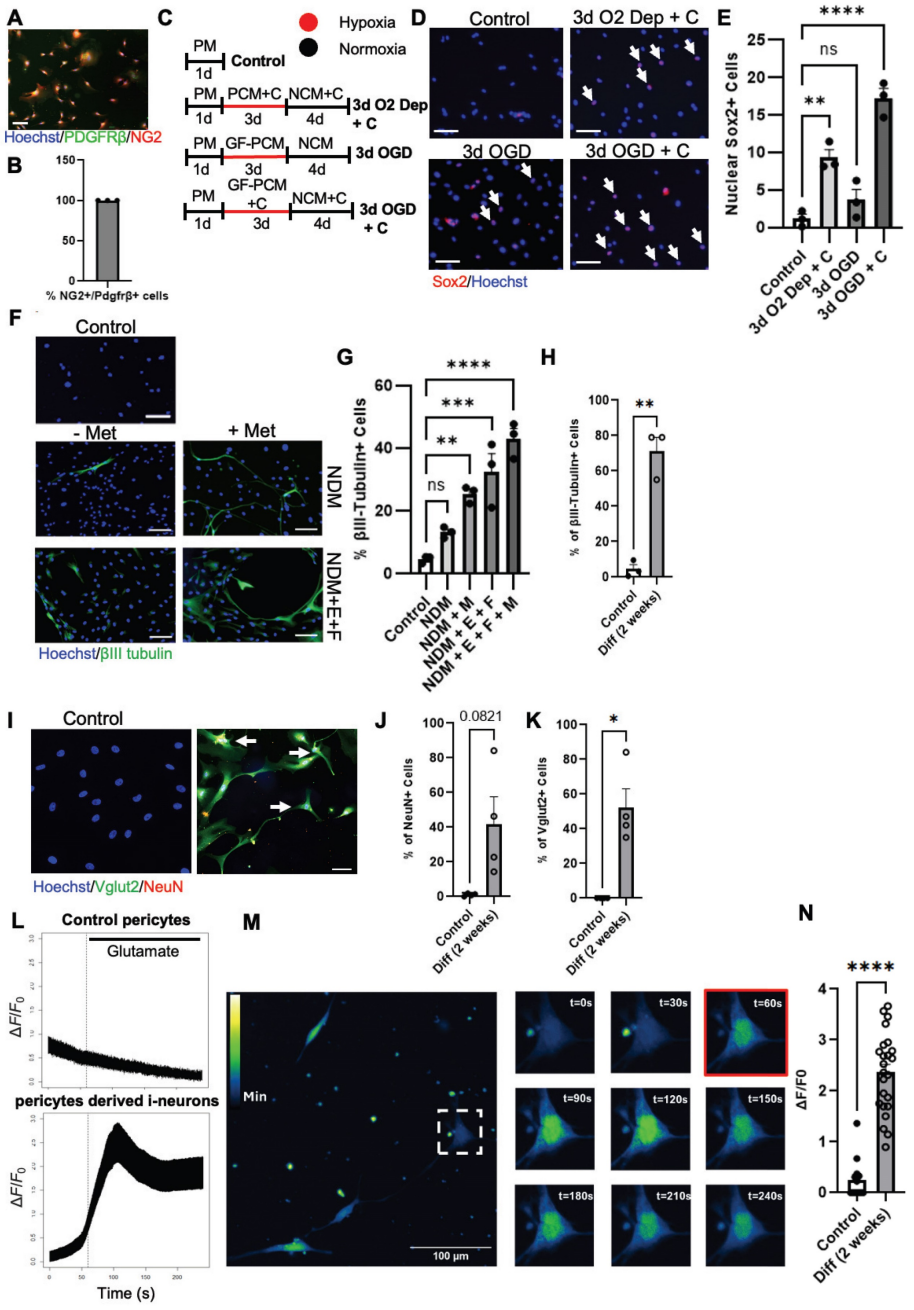
** Sequential treatment of CpdC and metformin facilitates reprogramming /differentiation of *NG2^+^* pericytes into functional neurons in culture.** (A-B) Representative image and quantification of cultured human pericytes isolated from human leptomeningeal tissues, immunostained for NG2 (red) and Pdgfrβ (green), counterstained with Hoechst (blue). (C) Experimental timeline of cultured human pericytes undergoing different conditions for neural reprogramming. (D-E) Representative images and quantification of the proportion of nuclear Sox2^+^ (red) i-NSCs over total live cells from either oxygen-glucose deprivation (OGD) or oxygen deprivation (O2 Dep) conditions. Experimental groups treated with CpdC were denoted with +C. Arrows denote Sox2^+^ i-NSCs, counterstained with Hoechst. (F-G) Representative images and quantification of the proportion of βIII tubulin^+^ (green) neurons over total live cells upon receiving NDM treatment in the absence and presence of EGF (E), FGF2 (F), and metformin (M) for 1 week, analysed with One-way ANOVA. (H) Quantification of the proportion of βIII tubulin^+^ neurons over total live cells upon receiving NDM + E + F + M treatment for two weeks, analysed with Student t-test. (I-K) Representative images and quantification of the proportion of Vglut2^+^ (green) and NeuN^+^ (red) neurons over total live cells upon receiving NDM + E + F + M treatment for two weeks, analysed with Student t-test, . Arrows denote Vglut2^+^/NeuN^+^ double-labeled neurons. n = 3-4 donor tissues per group. (L) Representative fluorescence traces of Fluo 4 AM before and after glutamate (10 µM) addition at 60 s from control pericytes and i-neurons derived reprogrammed pericytes. (M) Representative time-lapse fluorescent images of a single i-neuron labeled with Fluo 4 AM (glutamate addition, red). (N) Quantification of the amplitude of spikes in response to glutamate from control pericytes and i-neurons differentiated from reprogrammed pericytes. n=14 cells for control pericytes, and n=24 i-neurons from 3 donor tissues. Scale bar: 100 µm; ** P < 0.01, *** P < 0.001, **** P < 0.0001.

Since cortical pericytes and leptomeningeal pericytes are derived from the same embryonic origin of neural crest cells 17, we obtained leptomeningeal tissue from human patients. We then isolated primary NG2^+^/Pdgfrβ^+^ pericytes from the human leptomeningeal tissue for reprogramming cultures (Figure 8A-B). On the basis of murine cortical pericyte reprogramming culture conditions (Figure S11), we optimized neural reprogramming procedures for human pericytes. We found that CpdC treatment combined with oxygen-glucose deprivation (OGD with 1% O2 and glucose-free media) for 3 days (Figure 8C) induced the highest number of nuclear Sox2^+^ cells (Figure 8D-E) relative to a control group. Subsequently, we examined whether common neural coating substrates (poly-L-ornithine/laminin) can further enhance the reprogramming of human pericytes into Sox2^+^ i-NSCs. We found that poly-L-ornithine/laminin-coated plates with human pericytes under OGD + CpdC reprogramming process exhibited 38.8% nuclear Sox2^+^ i-NSCs, significantly higher than the un-coated plates (Figure S13A-C), while the same neural substrate coating under a control condition did not seem to change pericyte properties, as manifested by immunocytochemical analysis of pericyte markers Pdgfrβ and NG2 (Figure S13D). Together, these results reveal that OGD condition combined with CpdC treatment and neural substrate coating can maximally reprogram human pericytes into Sox2^+^ i-NSCs.

To further optimize conditions to produce newborn functional neurons from human pericytes, we cultured optimal reprogrammed human pericytes (receiving OGD + CpdC + neural substrate coating treatment) in NDM in the absence and presence of EGF (E), FGF (F), and metformin (M) for 1 week. We showed that the highest percentage of βIII tubulin^+^ neurons generated was in the NDM + E + F + M group, with 42.9% of cells expressing βIII tubulin (Figure 8F-G and Figure S13E). To test whether these βIII tubulin^+^ i-neurons can further develop into a mature stage to functionally respond to external neuronal stimulation, we extended the culture in NDM for 2 weeks and observed that 70% of cells expressed βIII tubulin and ~50% of cells were positive for Vglut2, a marker for an excitatory neuron subtype (Figure 8I, K). Interestingly, ~40% of the cells are also positive for NeuN, a mature neuronal marker (Figure 8I-J). We then performed calcium fluorescence imaging on these i-neuron cultures and found that they responded to glutamate stimulation to produce spikes, while control pericytes did not respond to the glutamate stimulation (Figure 8L-N).

Finally, we have produced human iPSC-derived neurons which were assessed after 2 weeks of differentiation 40. This serves as a positive control group for characterising human neuron morphology and functionality (Figure S14A-H). Interestingly, Vglut2 staining in 2-week-old human iPSC-derived neurons also showed a cytoplasmic pattern without classical puncta structure in cellular processes, which only appear in fully developed 7-12 weeks old mature neurons 41,42 (Figure S14I-J). This suggests that 2-week-old human neurons have a similar level of development regardless of their origin either from hiPSC or human pericytes. In addition, the 2-week-old human iPSC-derived neurons respond to glutamate treatment at the same level as 2-week human pericyte-derived neurons (Figure S14K-L).

## Discussion

The present study demonstrates that NG2^+^ pericytes have a unique and strong neurogenic potential to produce new neurons via a transient i-NSC stage following ischemic stroke under both *in vivo* and *in vitro* conditions. This reprogramming/differentiation process can be regulated by targeting the aPKC-CBP epigenetic pathway. Specifically, these findings support four major conclusions. First, we elucidate that naïve NG2^+^ and Tbx18^+^ pericytes are two distinct pericyte populations in the cerebral cortex by utilizing scRNA-seq. Second, we show that NG2^+^ pericytes have a strong neurogenic potential following ischemic stroke by generating RGPs, while Tbx18^+^ pericytes exhibit a strong potency to produce endothelial cells following ischemic stroke. Third, we demonstrate that CBP Ser436 dephosphorylation/phosphorylation modulates NG2^+^ pericyte reprogramming/differentiation process by regulating the acetylation shift between Sox2 and H2B and Sox2 nuclear-cytoplasmic trafficking. Fourth, we used an OGD-induced human pericyte reprogramming culture model to demonstrate that sequential treatment of CpdC and metformin facilitates reprogramming/differentiation of NG2^+^ human pericytes into functional neurons in culture.

Pericyte heterogeneity can be categorized by morphology, origin, and/or genetic marker diversity 2-5. In the current study, we used scRNA-seq, together with genetic lineage-traced animal models, to map cell atlases for both Tbx18^+^ and NG2^+^ pericytes. Here we reveal that Tbx18^+^ and NG2^+^ pericytes are two distinct subtypes of pericytes. Under the naïve condition, both canonical and activated canonical pericyte clusters possess 80% of NG2^+^ cells, while around 10% of NG2^+^ cells are smooth muscle cells in the cortex. The rest of the NG2^+^ cells are clustered as mesenchymal-like pericytes, endothelial cells, OPCs, and OLs. This is in line with previous studies using NG2 as a marker for pericytes, smooth muscle cells, and OPC/OL lineage cells 43-45. However, unexpectedly, NG2 labelled OPCs/OLs only occupy minimal amounts of the total NG2^+^ population in the cerebral cortex. This is possibly due to the grey matter (cerebral cortex) possessing much less myelinated axons and OPC/OL lineage cells when compared to the white matter. In addition, the endothelial cells present in the naïve NG2 sample suggests that there is an angiogenic potential of NG2^+^ pericytes, which has not been previously reported. On the other hand, in the naïve Tbx18 sample, the highest percentage of Tbx18^+^ cells are in the vascular-genic pericyte cluster, which barely shows up in the naïve NG2 sample. This unique vascular-genic pericyte cluster shows a direct trajectory to produce endothelial cells in the naïve Tbx18 sample, manifested by Monocle 3 pseudotime analysis with scRNA-seq. Intriguingly, canonical, activated canonical, and mesenchymal-like pericyte clusters are much smaller than the vascular-genic pericyte cluster in the naïve Tbx18 sample, while the percentage of smooth muscle cells in naïve Tbx18 sample remains the same as that in the naïve NG2 sample. Our scRNA-seq analysis provides a unique and complete cell atlas for Tbx18^+^ and NG2^+^ cells in the cerebral cortex and demonstrates that Tbx18 and NG2 mark two distinct pericyte populations, which are further confirmed by our immunohistochemical analysis.

Excitingly, our scRNA-seq analysis also provides compelling evidence showing that the two distinct pericyte populations respond differently to brain injury in terms of their cell plasticity. NG2^+^ pericytes can be reprogrammed into RGPs in response to injury with ischemic injury inducing the highest percentage of RGPs expressing Sox2, while Tbx18^+^ pericytes only generate minimal amounts of glial precursors in response to injury. This neural reprogramming capability was further confirmed by immunohistochemical analysis at the protein level, which showed that ischemic injury induced a higher number of Sox2^+^ i-NSCs and DCX^+^ neuroblasts from NG2^+^ pericytes than physical injury did. On the other hand, Tbx18^+^ pericytes produced minimal Sox2^+^ i-NSCs and DCX^+^ neuroblasts in response to injury. Since the *NG2-CreER^T2^* mouse line has been used to trace either pericytes or OPCs based on location and morphology 43,44,46 *in vivo*, it is extremely hard to address the origin of Sox2^+^ i-NSCs following brain injury while only using the linage tracing animal model. Here, our scRNA-seq approach is able to dissect pericytes and OPC lineage cells in different clusters from NG2 samples and subsequent single-cell pseudotime and velocity analysis emphasizes that it is the NG2^+^ canonical pericyte cluster that gives rise to RGPs but not OPC cluster, based on predicted trajectory direction following brain injury. Intriguingly, early work has shown that meningeal-derived RGPs traced by Pdgfrβ during the embryonic developmental stage contribute to postnatal neurogenesis in the cortex 47. Our current scRNA-seq analysis suggests that NG2^+^ pericytes, in response to severe brain injury such as ischemia, can recapitulate this embryonic developmental event to produce RGPs for local neurogenesis at the site of injury. However, Tbx18^+^ pericytes do not have the capability to produce neurons *in vivo*, which is consistent with previous reports showing that Tbx18^+^ pericytes cannot produce neurons at the lesion site following the cortical stab injury model 18.

Another difference between NG2^+^ and Tbx18^+^ pericytes is that NG2^+^ pericytes have no capability to produce fibroblasts and only moderately produce endothelial cells following both physical and ischemic injuries. In contrast, Tbx18^+^ pericytes show multipotency to produce fibroblasts and a large population of endothelial cells following brain injury. Pericytes contributing to fibrotic tissue formation following various central nervous system injured models have been well studied previously 15. Recent work also discloses the occurrence of Tbx18-derived fibroblasts following either a cortical stab injury model or a permanent focal ischemic stroke model 14,18. This is consistent with our current work using either a physical injury model with saline injections or a transient ischemic stroke model with ET-1 injections in the cerebral cortex. Our work clearly demonstrates that not all pericytes contribute to fibrotic tissue formation following injury. In addition, Tbx18^+^ pericytes, surprisingly, generated a large population of endothelial cells, occupying over 70% of Tbx18^+^ cells after ischemic injury. This result emphasizes its important role in remodelling vasculature following stroke. Tbx18^+^ pericytes seem to be biased to produce brain non-parenchymal cells, such as fibroblasts and endothelial cells, while NG2^+^ pericytes contribute to brain parenchymal cell genesis following injury. It is important to dissect such different roles of distinct pericyte populations following brain injury, with the ultimate goal of developing targeted therapeutic strategies to coordinate cellular reprogramming to promote optimal brain regeneration. One common feature of both NG2^+^ pericytes and Tbx18^+^ pericytes is their capability to produce microglia cells following brain injury, contributing to immunomodulation after injury. This seems to be a common phenotype alteration for all pericytes in response to brain injury since previous work using Rgs5 promoter-driven reporter mice reveals that Rgs5^+^ pericytes can be transdifferentiated into microglia after stroke as well 11.

Importantly, on the basis of our *in vivo* work, we developed a genomic integration-free methodology to reprogram pericytes into functional neurons using a hypoxia/glucose deprivation condition that mimics ischemic stroke *in vivo*. Hypoxia has been used to facilitate the reprogramming of somatic postmitotic cells into induced pluripotent stem cells 48,49. In the present study, we used hypoxia or OGD conditions to induce Sox2 accumulation in NG2^+^ primary murine/human pericytes *in vitro*. Recent work from human pericytes also shows that hypoxia can keep pericytes in a cell cycle stage without undergoing direct trans-neuronal differentiation 50. Another study discloses that direct reprogramming of adult human brain pericytes into functional i-neurons by ectopic expression of transcription factors, Ascl1 and Sox2, encloses a transient activation of a neural stem cell-like gene expression program that precedes bifurcation into distinct neuronal lineages 51. Our genomic integration-free methodology also involves the transient i-NSC stage to produce functional neurons derived from primary human pericytes, recapitulating the transcription factor(s)-induced pericyte neural reprogramming.

In addition, our current study elucidates the important role of CBP Ser436 dephosphorylation/phosphorylation in regulating pericyte neural reprogramming/differentiation by coordinating an acetylation shift between histone (H2B) and non-histone substrates (Sox2) to modulate Sox2 nuclear import/export. Both H2B and Sox2 are known direct targets of CBP 28-30. Our co-IP experiment in the SVZ NPC differentiation culture model supports that CBPS436 phosphorylation is required for metformin to enhance the CBP binding to Sox2 for acetylating Sox2, sequestering away from H2B (interaction and acetylation). Therefore, we reasoned that the competing model between Sox2 and H2B for CBP binding is controlled by CBP S436 phosphorylation: phosphorylated CBP Ser436 favors CBP binding to Sox2 over H2B. We propose that the substrate competing model for CBP phosphorylation is underlying molecular mechanisms for acetylation shift between Sox2 and H2B. However, this sequestration model will need to be more thoroughly addressed in the future. We also demonstrate that CBP Ser436 dephosphorylation/phosphorylation can be controlled by pharmacological reagents modulating an energy sensor, AMPK, activity to facilitate i-NSC reprogramming from pericytes and their subsequent differentiation into neurons. Therefore, our discovery provides fundamental knowledge for the development of pharmacological strategies that can promote local neural regeneration at the site of injury by sequential application of CpdC and metformin treatment to not only enhance cellular reprogramming of a-pericytes into i-NSCs but also promote the neuronal differentiation of these i-NSCs to generate neurons (Figure S10C). Future studies using a rodent stroke model will validate the neural regenerative potential of these pharmacological strategies employed locally at the site of injury by targeting a-pericyte cellular reprogramming/differentiation. Particularly, increasing studies show that ischemia-activated pericytes possess the capabilities to be reprogrammed into NSCs *ex vivo* and *in vivo* in both rodent stroke models and human stroke patients 6,7,16. As efforts are made to translate this new concept into clinical application, our findings pave the road for developing novel pharmacological approaches to enhance local neuronal regeneration from a-pericytes as an autologous cell source at the site of stroke-related brain injury.

In summary, we revealed that two distinct subtypes of pericytes possess different reprogramming potencies in response to physical and ischemic injuries. NG2^+^ pericytes exhibit strong neural reprogramming potential, while Tbx18^+^ pericytes display strong vascular-genic potency. In addition, this study elucidates underlying mechanisms through which the aPKC-CBP pathway regulates NG2^+^ pericyte neural reprogramming and differentiation by coordinating acetylation between Sox2 and H2B to control Sox2 nuclear import/export. Finally, we demonstrate that sequential treatment of CpdC and metformin by targeting the aPKC-CBP pathway facilitates OGD-induced reprogramming/differentiation of human NG2^+^ pericytes into functional neurons in culture.

## Methods

### Animals

All animal use was approved by the Animal Care Committee of the University of Ottawa in accordance with the Canadian Council of Animal Care policies. All the mouse lines, *Tbx18-CreER^T2^/Ai14-flx*, *NG2-CreER^T2^/Ai14-flx*, *CbpS436A*, *Sox2-GFP*, and *B6129SF2/J* mice were maintained on a 12 h light/12 h dark cycle with ad libitum access to food and water. Only wild-type (WT) and homozygous (*CbpS436A*) mice 52 were used as experimental mice and heterozygous of *CbpS436A* were used for breeding. We used both male and female B6129SF2/J mice (#101045, The Jackson Laboratory) for primary pericyte culture experiments. We used both male and female *Tbx18-CreER^T2^* mice [Tbx18tm3.1(cre/ERT2)Sev/J, The Jackson Laboratory, #031520, RRID:IMSR_JAX:031520] crossed to Ai14 tdTomato (tdT) reporter line [B6.Cg-Gt(ROSA)26Sortm14(CAG-tdTomato)Hze/J, strain #007914, RRID:IMSR_JAX:007914], and *NG2-CreER^T2^* mice [B6.Cg-Tg(Cspg4-cre/Esr1*)BAkik/J, The Jackson Laboratory, #008538, RRID:IMSR_JAX:008538] crossed to Ai14 tdT reporter line for scRNA-seq experiments and immunohistochemical experiments. The Sox2-GFP reporter transgenic mice were obtained from The Jackson Laboratory (B6;129S-Sox2tm2Hoch/J, #017592) for primary pericyte culture experiments. Each set of experiments was performed with littermates.

### ET-1/L-NAME surgery

Mice (2-4 months old) were anesthetized using 4-5% isoflurane and 1.5% oxygen and mounted to a stereotaxic frame for ET-1/L-NAME injections. Injections were performed using a Hamilton 10 µL gastight syringe with a 0.49 mm diameter needle (Hamilton Robotics, Reno NV, 7653-01). Injections of saline, or ET-1 (Abcam, Cambridge, UK, AB120471) (2 µg/µL) + L-N^G^- Nitroarginine methyl ester (L-NAME) (Sigma-Aldrich, St. Louis MS, N5751) (2.7 µg/µL) were performed at +0.0mm anterior-posterior (AP), -2.0mm Medial-Lateral (ML), -1.6mm Dorsal-Ventral (DV); and +0.2AP, -2.0ML, -1.4DV. ET-1 and L-NAME were dissolved in phosphate-buffered saline (PBS) and sonicated in a 4^o^C water bath for 15 min before use. The injection was performed at 0.2 µL/min for 5 min per injury site for a total of 1 µL/injection. Upon needle insertion, a 1 min waiting time was used to allow for the settling of tissue. Following injection, a 3 min waiting time before needle removal was used to reduce back-flow. Body temperatures were continually monitored and maintained at 37^o^C during surgery using a heating pad and anal thermometer. Local subcutaneous Bupivacaine (s.c. 0.05 mg/kg) was given once at surgery and then 4-6 h later. Transdermal bupivacaine 2% was administered post-surgery over the incision at closure. All animals that received stroke were included in the study.

### scRNA-seq

#### Sample preparation for ScRNA-seq

We performed scRNA-seq using ET-1/L-NAME stroke-injured cerebral cortical tissues isolated from two pericyte lineage tracing mice (*NG2-CreER^T2^/Ai14-flx* and* Tbx18-CreER^T2^/Ai14-flx*) that received intracerebral injections of ET-1/L-NAME (or saline) 3 days prior to scRNA-seq. Both transgenic mice were also treated with tamoxifen 7 days before injury to enable lineage tracing of *Tbx18-Ai14^+^* and *NG2-Ai14^+^* cells. Three groups of *Tbx18-Ai14^+^* and *NG2-Ai14^+^* cells from 1) no injury (naïve), 2) physical injury (saline), and 3) ischemic injury (ET-1/L-NAME) were fluorescence-activated cell (FAC) sorted for tdT (Ai14)^+^/DAPI^-^ live cells to perform scRNA-seq (Figure 1A and Figure S1A). Naïve cortical tissues from two hemispheres of 6 animals and injured cortical tissues from 10-12 animals (equal numbers of female and male tissues were included) were dissected and collected for papain digestion. The minced cortical tissues were incubated in 150 µL papain solution (2 mg/mL, Worthington Biochemicals, LS003126), together with 100 units DNase (Sigma-Aldrich, D5025-15KU), at 37^º^C for 30 min in 360° HulaMixer^TM^ Sample Mixer (Thermo Fisher, 15920D). After the first 15 min, the tissues were triturated 15 times using a P1000 pipette. At the end of 30 min, papain solution was removed by centrifugation and cell pellets were resuspended in 250-500 μL of Pericyte Conditioned Medium (PCM) containing high glucose Dulbecco's Modified Eagle Medium (DMEM) (Wisent Bioproducts, 319-005-CL)/F-12 (Thermo Fisher, 11765-054) supplemented with 2% fetal bovine serum (FBS) (Life Technologies, 12484010), 5 µg/mL epidermal growth factor (EGF) (VWR, CACB354052), 5 µg/mL fibroblast growth factor (FGF) (Peprotech, 100-18B), 1% N2 supplement (Thermo Fisher, 17502048) and 0.33% Penicillin-Streptomycin (Thermo Fisher, 15140122). The tissues were further homogenized by passing through a 21G needle and a 23G needle, sequentially. Subsequently, the homogenized tissue samples were carefully overlayed on 22% Percoll (Sigma-Aldrich, P1644) and centrifuged at 560 g at 4°C for 10 min to purify the healthy cell pellets. The healthy cell pellets were resuspended in 500 µL DAPI solution and sent for FAC sorting (FACS core, OHRI). The sorted Ai14 (tdTomato, tdT)^+^/DAPI^-^ cells per group (Figure 1A and Figure S1A) were collected in Hanks' Balanced Salt Solution (HBSS)/ bovine serum albumin (BSA) buffer. The final concentration of cell suspension was around 10,000-15,000 cells/10 µL.

#### ScRNA-seq library preparation and sequencing

The scRNA-seq library preparation and sequencing were performed at StemCore laboratories from the OHRI. The FAC-sorted cell suspensions per group were submitted for single cell 3' RNA-Seq using 10X Genomics Chromium (10X Genomics, Pleasanton, CA, 1000204) and Illumina next-generation sequencing technologies. (Illumina Nextseq 500 Sequencer).

The sorted cells were quantitated and checked for viability assessment using the Countess II cell counter (Thermo Fisher, I-CACC2). Following cell counting and viability assessment, the suspended cells were loaded on a Chromium controller Single-Cell instrument to generate Barcoded single-cell Gel Bead-In-Emulsions (KIT# 1000121). GEMs were broken and the barcoded cDNA was amplified using the C1000 Touch Thermal cycler with 96-Well Reaction Module (Thermal cycler, 1851196). The amplified barcoded cDNA was then fragmented, A-tailed and ligated with adaptors (KIT# 1000121.). Finally, PCR amplification was performed to enable sample indexing and enrichment of the 3′ RNA-Seq libraries (Single Index Kit T, 1000213). The final libraries were quantified using a Thermo Fisher Qubit dsDNA HS Assay kit (Q32854) and the fragment size distribution of the libraries was determined using the AATI Fragment analyzer High Sensitivity NGS kit (DNF-486-0500). Pooled libraries were then sequenced using Illumina Nextseq. All samples were sequenced to approximately 20,000 reads per cell for V3.1 Kit (1000121) as per 10x Genomics recommendation. The resulting average of 3143 cells/group had an average of 2567 unique genes detected and 6504 UMI counts per cell.

#### Processing of raw sequencing reads

Raw sequencing reads were processed using cellranger v4.0.0-v7.0.0 and the mm10 build of the mouse genome.

#### ScRNA-seq analysis

The expression matrix for each of the 6 conditions (*NG2-tdT^+^* naïve, *NG2-tdT^+^* physical injury, *NG2-tdT^+^* ischemic injury, *Tbx18-tdT^+^* naïve, *Tbx18-tdT^+^* physical injury, *Tbx18-tdT^+^* ischemic injury) was loaded into R as a Seurat (v4.3.0.1) object. The datasets were stored in separate objects. Each Seurat object was filtered to contain only genes present in a minimum of 3 cells and cells containing a minimum of 200 genes detected. Data quality control was then performed on each Seurat object separately to remove 1) cells with genes and RNA counts +/- 2 standard deviations outside of the mean, 2) cells with greater than 12% mitochondrial gene transcripts, and 3) genes "Gm42418" and "AY036118". These were removed from the datasets as they have been previously reported as rRNA contamination 53,54. The datasets were then integrated into three combinations of the six Seurat objects 1) *NG2-tdT^+^* naïve and *Tbx18-tdT^+^* naïve; 2) *Tbx18-tdT^+^* naïve, *Tbx18-tdT^+^* physical injury, and *Tbx18-tdT^+^* ischemic injury; and 3) *NG2-tdT^+^* naïve, *NG2-tdT^+^* physical injury, and *NG2-tdT^+^* ischemic injury. A fourth combination of Seurat objects was created which combined *NG2-tdT^+^* naïve, *Tbx18-tdT^+^* naïve, and a publicly available dataset, *Tbx18-tdT^+^* sham, from Pham *et al.*, 2021 33. Additionally, Integration features were identified by the top 3000 variable genes, and integration anchors were identified using the Canonical Correlation Analysis (CCA). The datasets were integrated using the identified anchors and the “SCT” normalization method. Principal component analysis (PCA) was performed on the highly variable genes, and Uniform Manifold Approximation and Projection (UMAP) embeddings were calculated from the first 17, 15, 13, and 22 principal components with 0.3, 0.1, 0.4, and 0.4 resolution for the four integrated datasets, respectively. Before conducting downstream analyses, we scaled and normalized the data using the ScaleData and NormalizeData functions. Clusters were identified by the expression of known cell-type markers utilizing the FindAllMarkers function and specifying a log fold-change threshold > 0.25.

### Gene Ontology (GO)

Gene Ontology (GO) annotations were analyzed using clusterProfiler (v4.8.3) specifying a log fold-change threshold > 0.25 and a p-value adjusted < 0.05.

### RNA velocity and Monocle3

PCA and UMAP embeddings that were produced during the initial analysis step were embedded using Seurat. Velocity estimates were calculated using the scVelo (v0.2.2.) python package with default parameters. Velocity vectors and velocity graphs were computed and then projected on UMAP embeddings.

Pre-processed scRNA-seq data using Seurat were converted into an object compatible with Monocle3 (v1.3.1). The root state argument was called and defined as the branch containing pericyte markers.

### Ischemia-activated pericyte culture and sphere formation

Mice were sacrificed to remove their brains 3 days following ET-1/L-NAME surgery. The infarct/peri-infarct cortical tissues were micro-dissected in Minimum Essential Medium (MEM) and digested (Thermo Fisher, 12360038) for 30 min using 150 µL papain (2 mg/mL, Worthington Biochemicals, LS003126) and 100 units DNAse (Sigma-Aldrich, D5025-15KU) at 37ºC. The samples were then triturated through 21- and 23-gauge needles. Single dissociated cells were plated at 600,000 cells/mL in an uncoated twelve-well plastic dish with PCM: DMEM (Wisent Bioproducts, 319-005-CL)/F-12 (Thermo Fisher, 11765-054) containing 0.33% Penicillin-Streptomycin, 5 µg/mL EGF (VWR, CACB354052), 5 µg/mL FGF (Peprotech, 100-18B), 1% N2 supplement (Thermo Fisher, 17502048), and 2% FBS (Life Technologies, 12484010). Full media changes were performed on days 1 and 2 post-plating and half media changes every other day following that.

After 1-2 weeks, ischemia-activated pericytes (a-pericyte) were selectively grown and became fully confluent, showing 100% population of cells expressing pericyte markers, Pdgfrβ and NG2 (Figure 6A). These a-pericytes were exposed to Neural Conditioned Medium (NCM): 50% DMEM/F-12 (Thermo Fisher, 11330032) +50% neural basal media (Thermo Fisher, 21103049) containing 0.33% Penicillin-Streptomycin, 5 µg/mL FGF, 5 µg/mL EGF, 10 µg/mL leukemia inhibitory factor (LIF) (Peprotech, 250-02), and 1% N2 with and without CpdC (5 µM, Calbiochem, 171260) for 2-4 weeks. 200 µL of media was added once a week to account for evaporation. Single spheres were collected onto glass slides using a Cytospin (Thermo Fisher) between 2 and 4 weeks. Single spheres were also picked 4 weeks after NCM treatment using a p200 pipette and placed into a dish coated with Matrigel (Fisher Scientific, CB-40230C) in Neural Basal Media containing 0.33% Penicillin-Streptomycin with and without metformin (1 µM, Sigma-Aldrich, PHR1084) treatment. Following 2 days, the differentiating i-NSCs were immunostained for Sox2 and GAPDH to assess nuclear-cyto trafficking. After 7 days, another set of differentiating i-NSCs were immunostained for βIII tubulin to assess neuronal differentiation. Metformin (Sigma-Aldrich, PHR1084) stock solution (1mM) was prepared in 1x PBS and CpdC (EMD Millipore, 171260) stock solution (5mM) was prepared in Dimethyl sulfoxide (DMSO).

### SVZ neurosphere culture and transfection

For adult subventricular zone (SVZ) NPC cultures, the subependymal of the lateral ventricles were dissected according to previously published work 27. Cell density and viability were determined via trypan blue exclusion and a hemocytometer. Freshly isolated cells were then plated at a density of 10 cells/µL in six-well plates (Thermo Fisher Scientific, 12-556-004) in serum-free medium (SFM) containing 20 ng/mL EGF (Sigma-Aldrich, E9644), 20 ng/mL FGF (PeproTech, 100-18B), 1x B-27™ supplement (Thermo Fisher Scientific, 17504-044) and 2 µg/mL heparin (Sigma-Aldrich, H3149-100KU). Neurospheres were collected every 7 days and passaged at a density of 2 cells/µL in six-well plates.

For differentiation, secondary neurospheres were collected, gently triturated, and plated onto poly-L-ornithine (Sigma-Aldrich, P4957) and laminin (Thermo Fisher Scientific, CB40232)-coated six-well plates at 1,000,000 cells/well in Serum-Free Media (SFM) , supplemented with 10% FBS in the absence (PBS) or presence of metformin (1 μM) (Sigma-Aldrich, PHR1084) for 7 days. The differentiating NPCs were then collected by gentle scraping and used for immunoprecipitation and western blot analyses as described below.

To assess endogenous Sox2 nuclear/cytoplasmic trafficking, secondary neurospheres were grouped, triturated, and plated onto poly-L-ornithine and laminin-coated coverslips in 24-well plates at 100,000 cells/well in SFM supplemented with 10% FBS in the absence (PBS) or presence of metformin (1 μM) for 6 days. The differentiating NPCs were treated with MG132 (1 μM) (Sigma-Aldrich, M7449) for 16 h prior to fixation. For exogenous Sox2 trafficking, secondary neurospheres were grouped, gently triturated, and plated onto poly-L-ornithine and laminin-coated coverslips in 24-well plates at 100,000 cells/well in SFM containing 20 ng/mL EGF, 20 ng/mL FGF2, 1x B-27™ supplement, and 2 µg/mL heparin. Then, 0.6 μg pLPS-hSox2-GFP plasmid (Addgene, 49390) and 1.8 μl TransIT-X2 (Mirus, MIR6004) were mixed with 50 μl DMEM/F12 medium, incubated for 30 min and added to NPCs 24 h after plating. 24 h post-transfection, NPCs were directed to differentiate in the presence of SFM supplemented with 10% FBS and either metformin (1 μM) or PBS for 3 days. The differentiating NPCs were treated with MG132 (1 μM) for 16 h prior to fixation. Metformin stock solution (1 mM) was prepared in 1x PBS and MG132 (Sigma-Aldrich, M7449) stock solution (1 mM) was prepared in DMSO. The drugs were added to the cultures at various stages of cultures as indicated.

### Mouse pericyte culture

2-4-month-old B6129SF2/J adult mice were sacrificed to remove their brains. The cerebral cortical tissues were collected and minced in MEM and digested for 30 min using 150 µL papain and 100 units DNAse at 37ºC on a 360° HulaMixer^TM^ Sample Mixer (Thermo Fisher, 15920D). The digested samples were centrifuged at 1500 rpm for 5 min and cell pellets were then resuspended in 250 µL PCM. The resuspended cells were forced to pass through 21- and 23-gauge needles twenty times to dissociate the cells at the single-cell level. Then, 1.7 volumes of 22% BSA (sterile, prepared in PBS) were added and mixed well. The samples were centrifuged at 4000 rpm for 10 min and the cell pellets were washed once with PCM medium followed by centrifugation at 1500 rpm for 5 min. Finally, the resuspended single-dissociated cells were plated in an uncoated twelve-well plastic dish with PCM to selectively culture murine pericytes. Full media changes were performed on days 1 and 2 post-plating and half media changes every other day following that.

The passage 2-4 of cultured murine pericytes were used for experiments. For experiments, murine cortical pericytes were seeded in 24-well plates (control, middle reprogramming, and differentiation stages) at 12,000 cells per well in Pericyte Medium (PM): DMEM/F-12 containing 0.33% Penicillin-Streptomycin, 5 µg/mL EGF, and 2% FBS. On the next day, the control plate under normoxia with PM medium was fixed with 4% paraformaldehyde (PFA). Other wells were then transferred to a hypoxia chamber set to 3% O2 and 5% CO2 for 5 days (Figure S11A). two medium conditions were tested for neural reprogramming efficiency: (1) PCM, (2) PCM + CpdC (5 µM) (Figure S11A). 5 days later, the middle reprogramming stage plate was retrieved from hypoxia. The media was switched to NCM or NCM + CpdC (5 µM), and the plates were incubated in normoxia for 4 days (Figure S11A). After 4 days, the middle reprogramming stage plate was fixed with 4% PFA. Finally, to examine neuronal differentiation of reprogrammed induced-neural stem cells (i-NSC) from cortical pericytes, the hypoxia-reprogrammed murine pericytes were continually cultured in the NCM only medium for 2 days to wash out CpdC effects (Figure S11A) and subjected to neuronal differentiation in the presence neuronal differentiation medium (NDM): 50% DMEM/F-12 +50% neural basal medium containing 0.33% Penicillin-Streptomycin, 10 µg/mL LIF (PeproTech, 250-02), 1% N2 (Thermo Fisher, 17502048), B27 (Thermo Fisher, 17504-044), Retinoic acid (RA, Sigma-Aldrich, R2625), and BDNF (PeproTech, 450-02) (Figure S11A). Three different conditions were tested under differentiation stage: (1) absence of CpdC and metformin; (2) absence of CpdC in the reprogramming stage but presence of metformin (1 µM) in the differentiation stage; (3) presence of CpdC (1 µM) in the reprogramming stage and metformin (1 µM) in the differentiation stage for 7 days before fixation (Figure S11A).

2-4 month old *NG2-CreER^T2^/Ai14-flx* and *Tbx18-CreER^T2^/Ai14-flx* adult mice were sacrificed to remove their brains. The cerebral cortical tissues were collected and pericytes were selectively cultured as previously described. Murine cortical pericytes derived from both mice lines were seeded in 24-well plates at 12,000 cells per well in PM. *In vitro* Ai14 expression was induced by 1 µM 4-Hydroxy-Tamoxifen (EMD Millipore, 508225) added to the culture media for 1-2 days. Following 4-Hydroxy-Tamoxifen, the cultured pericytes were undergone reprogramming and neuronal differentiation process as described above.

### Human pericyte culture

The use of human surgical leptomeningeal tissues was approved by the Ottawa Health Science Network Research Ethics Board. Briefly, human leptomeningeal tissue was transported in cold HBSS from the operating room back to the laboratory. First, the tissues were cut and minced into small pieces in the MEM media. Then, the small pieces of tissues were triturated 20 times using a P1000 pipette and centrifuged at 1500 rpm for 5 min. Following careful removal of the supernatant, the cell pellets were resuspended in 300-500 μL of PCM. The resuspended cells were then forced to pass through 18- and 23-gauge needles to dissociate the cells at the single-cell level. Then, 1.7 volumes of 22% BSA (sterile, prepared in PBS) were added and mixed well. The samples were centrifuged at 4000 rpm for 10 min and the cell pellets were washed once with PCM media followed by centrifugation at 1500 rpm for 5 min. Finally, the resuspended single-dissociated cells were plated in an uncoated twelve-well plastic dish with PCM to selectively culture human pericytes from the leptomeningeal tissues. The half a media change was performed every 3 days until reaching confluence.

The passage 2-6 of cultured human pericytes were used for experiments. For experiments, human pericytes were seeded in 24-well plates (control, reprogramming and differentiation stages) at 4000-8000 cells per well in PM: DMEM/F-12 containing 0.33% Penicillin-Streptomycin, 5 µg/mL EGF, and 2% FBS. On the next day, the control plate under normoxia with PM medium was fixed with 4% PFA. The rest wells were then transferred to a hypoxia chamber set to 1% O2 and 5% CO2 for 3 days (Figure 8C). three media conditions were tested for neural reprogramming efficiency: (1) PCM + CpdC (5 µM), (2) PCM (with glucose-free DMEM (GF-DMEM) (Thermo Fisher 11966-025)), (3) PCM (with GF-DMEM) + CpdC (5 µM) (Figure 8C). 3 days later, the middle reprogramming stage plate was retrieved from hypoxia. The media was switched to NCM or NCM + CpdC (5 µM), and the plates were incubated in normoxia for 4 days (Figure 8C). After 4 days, the middle stage plate was fixed with 4% PFA. In addition, to examine the effect of coating materials on human pericyte neural reprogramming efficiency, pericytes were seeded onto two 24-well plates coated with 0.01% poly-L-ornithine (Sigma-Aldrich P4957) and 5% laminin (Thermo Fisher CB40232). The plates were separated based on the control and reprogramming stages (Figure S13A). On the day of oxygen-glucose deprivation (OGD, 1% O2 and glucose-free PCM (GF-PCM), the control plate was fixed with 4% PFA. The reprogramming stage plate was then transferred to a hypoxia chamber set to 1% O2 and 5% CO2 for 3 days in the presence of GF-PCM + CpdC (5 µM) (Figure S13A). 3 days later, the reprogramming stage plate was switched to NCM + CpdC (5 µM) and incubated in normoxia for 4 days before fixation.

To examine neuronal differentiation of reprogrammed i-NSC from human pericytes, the cultured human pericytes were seeded onto 24-well plates coated with 0.01% poly-L-ornithine and 5% laminin. The control plate was fixed with 4% PFA before OGD started. Other plates were first transferred to a hypoxia chamber set to 1% O2 and 5% CO2 for 3 days in the presence of GF-PCM + CpdC (5 µM) (Figure S13E), and then incubated in normoxia for 4 days in the presence of NCM + CpdC (5 µM) followed by a two-day treatment in the presence of NCM only to wash out CpdC (Figure S13E). Subsequently, the reprogrammed human pericytes were subjected to four differentiation conditions (1) NDM, (2) NDM + 0.1% epidermal growth factor (E) and 0.1% fibroblast growth factor (F), (3) NDM + metformin (10 µM), and (4) NDM + 0.1% EGF and FGF2 + metformin (10 µM) for 1week or 2 weeks before fixation.

### Human iPSCs derived NSCs and neurons

#### Human iPSC culture

Human induced Pluripotent Stem Cell line was obtained from The Ottawa Human Pluripotent Stem Cell Facility (HPSCF) and maintained on diluted Matrigel (BD Biosciences, 354230) in mTeSR Plus media (STEMCELL Technologies, 100-0276). Media was replaced and iPSC passaged every 4-5 days using 0.5 mM EDTA.

#### Neural induction

hiPSCs were maintained as intact colonies on Matrigel and neural induction was initiated when cultures reached 90-100% confluence using two small molecular inhibitors, namely SB431542 (SB 10 µM) and LDN-193189 (LDN 500 Nm) 40. mTeSR media was replaced by KSR media containing both SB and LDN and changed daily from day 0-3. KSR media is composed of Knockout DMEM (Thermo Fisher Scientific, 10829018), 15% Knockout-serum replacement (Thermo Fisher Scientific, 10828028), 1x Glutamax, 1x MEM-NEAA (Thermo Fisher Scientific, 11140050), 0.1% β-ME (Thermo Fisher Scientific, 21985023), 1x Gentamicin and 1x Penicillin-Streptomycin.

At day 4 N2 media containing LDN was added in increasing amounts following ratios (KSR:N2): days 4-5 (3:1), days 6-7 (1:1), day 8-9 (1:3), days 10-11 100% N2 media and NSCs were harvested at day 12. LDN was supplemented all days while SB only from day 0-3. N2 media is composed of DMEM/F12, 0.15% glucose, 0.5x N2 supplement, 5 µg/mL human Insulin, 5mM HEPES (Thermo Fisher Scientific, 15630080), 1x Gentamicin and 1x Penicillin-Streptomycin. Cells were maintained at 37°C, 5% CO2 during differentiation.

#### HiPSC-NSC culture

Human iPSC derived Neural Stem Cells (hiPSC-NSC) were also maintained on diluted Matrigel in neural induction media (NIM) containing 1:1 DMEM/F12: Neuro Basal Media (ThermoFisher Scientific, 11320033, 21103049), 0.5x N2 Supplement (Thermo Fisher Scientific, 17502048), 0.5x B27 Supplement (Thermo Fisher Scientific, 17504-044), 0.5x Glutamax (Thermo Fisher Scientific, 35050061), 5 µg/mL human Insulin (Wisent Bioproducts #511-016-CM), 0.02 µg/mL FGF2 (Thermo Fisher Scientific, PHG0261), 0.02 µg/mL hEGF (Sigma, E9644), 1x Gentamicin (Wisnet Bioproduct, 450-135) and 1x Penicillin-Streptomycin (Thermo Fisher Scientific, 15140122). For passaging, NSCs were supplemented with 10 µM ROCKi (y-27632; Tocris, 1254) 2 h before lifting and up to 24 h post plating. The cells were dissociated using Accutase (STEMCELL Technologies, 07920) and plated at a density of 100,000 cells/cm^2^. Media was changed every 3 days and cells were passaged when confluent typically 1 week for a maximum of 10 passages.

#### Neuronal differentiation of hiPSC-NSCs

hiPSC-NSCs were treated with 10 µM ROCKi before harvesting with Accutase and plated on poly-L-ornithine/laminin-coated glass coverslips in 24 well plates at 40,000-60,000 cells per well in NIM media +ROCKi. Media was replaced next day to remove ROCKi and start neuronal differentiation using Neuronal differentiation media (NDM), containing 1:1 DMEM/F12: Neuro Basal Media, 0.5x N2 supplement, 0.5X B-27 supplement, 20 ng/mL each of BDNF (Peprotech, 450-02) and GDNF (Peprotech, 450-10), 200nM L-ascorbic acid (FujiFim Wako Chemicals, 323-44822), 1x Gentamicin and 1x Penicillin-Streptomycin. Media was changed every 3 days and cells were fixed at day 6 and day 15 after initiating differentiation for immunostaining.

### Calcium imaging

Human pericytes and human iPSC-derived NSCs were cultured and differentiated into neurons in 4-well glass bottom (ibidi) chambers coated with poly-L-ornithine and laminin for live imaging. Human cells were incubated in Tyrode's solution (mM: 124 NaCl, 5 KCl, 2 CaCl2, 1 MgCl2, 30 glucose, 25 HEPES; pH 7.4) loaded with 1 µM Fluo-4-AM for 30 min (from ThermoFisher/Molecular Probes, Waltham, MA). After acquiring a 10-30 s base line with 488 nm laser, cells were stimulated with glutamate 10-100 µM simultaneously with the image acquisition. Fluorescence signals were recorded at 18 frames/s for 4 min collected on an inverted Olympus XI-71 GE DeltaVision Elite microscope using a x20, 0.75NA objective (Nikon PLS Apo) and a D-F-T-C polychroic filter cube. Images were collected using Scientific CMOS Camera with 1x1 binning. All measurements were made in an environmental control chamber with temperature of 35.8℃. Associated software SoftWoRx with integrated Resolve3D were utilized for image acquisition design.

Intensity Fluorescence Analysis using the Fiji distribution of ImageJ software 55. All fluorescence signals were expressed as (Fmax - F0)/F0, where Fmax is the maximal fluorescence intensity and F0 the mean intensity of 10 frames recorded before the stimulus, both recorded in the region of stimulation. The maximum signal amplitude, Fmax, was estimated by identifying the maximum intensity value induced by the stimulus.

### Tissue preparation and immunohistochemistry

Mice were anesthetized with 0.1 ml of pentobarbital via intraperitoneal injection at the sacrificing day. Subsequently, the mice were perfused with cold 1x PBS, followed by cold 4% PFA (Sigma-Aldrich, 158127). Brains were then extracted and placed in 4% PFA overnight and dehydrated in a solution of 30% sucrose for 48 h. Brains were embedded in optimal cutting temperature compound (OCT) (VWR, 95057-838) and flash frozen on dry ice. 20 μm thick sections of the cortical region were cut using a cryostat (Leica Biosystems, CM1850) and collected on microscope slides (Fisher, 12-550-15). Slides were stored at -80^o^C until used for immunohistochemistry.

Frozen sections were defrosted in a dry incubator at 37^o^C for 10 min, followed by a 10-min incubation in 4% PFA. Sections were then washed three times with 1x PBS for 5 min each. Antigen retrieval was performed for the following primary antibodies: goat anti-Pdgfrβ, rabbit anti-Tbx18, and rabbit anti-NG2, by submerging the tissue sections in a 10mM Sodium Citrate solution pH 6.0 at 95^o^C for 6 min before permeabilization and blocking. Tissue permeabilization and blocking was performed using either 10% normal goat serum (NGS) or 3% BSA in PBS-T (0.3% Triton-100 in 1x PBS) and incubated for 1 h at room temperature. Primary antibodies were diluted in blocking buffer and incubated overnight at 4^o^C in a humidified chamber.

On the second day, sections were washed with PBS-T three times, 5 min each, and incubated in secondary antibodies diluted in PBS-T for 1 h at room temperature. After secondary antibody incubation, sections were counterstained with Hoechst 33342 (Cell Signaling Technology, 4082) at 1:1000 in PBS-T for 5 min and then washed three times with PBS-T for 5 min each. Slides were mounted with glass coverslips using PermaFluorTM mounting medium (Thermo Fisher Scientific, TA-030-FM) and dried overnight at room temperature.

### Immunocytochemistry

For immunocytochemistry, the cultured cells were fixed with 4% PFA, blocked, and permeabilized with 10% normal goat serum (NGS, Invitrogen, 10000C) or 3% BSA in PBS-T (0.3% Triton-100 in 1x PBS). Fixed cells were then incubated with primary antibodies at 4ºC overnight, with secondary antibodies at room temperature for 1 h, counterstained with Hoechst 33342 (1:1000, Sigma-Aldrich) and mounted with Permafluor (Thermo Fisher TA-030-FM).

### Imaging and cell quantification

For brain sections, fluorescent imaging was performed using a Zeiss Image M2 fluorescent microscope with Zeiss Zen Blue software (Oberkochen, Germany). For each mouse the entire injury lesion was captured. Dependent on the size of the injury, between 3 and 5 coronal sections were sampled every 10 series of sections for imaging. 1-3 images per section were captured in the Z-axis at a maximum of 1 µm apart and processed as an optical stack of 6-10 scanned slices for quantification.

For immunocytochemistry, digital image acquisition was performed using either a Zeiss Axioplan 2 fluorescent microscope with Zeiss Axiovision software that contains z-axis capability, or a Zeiss LSM 800 confocal microscope using Zeiss Zen software V2.0 (Oberkochen, Germany). 6-10 images were captured in the Z-axis at a maximum of 1 µm apart and processed as an optical stack of 6-10 scanned slices for quantification.

For quantification, Fiji software was used for cell quantification and image processing. Images were obtained at 20x magnification. At least 30-50 cells from individual experiments for each sample were quantified for the Sox2 nuclear-cytoplasmic intensity ratio using CellProfiler software (Broad Institute, MA). Here, Hoechst staining was used to define the boundary of the nuclear membrane and GAPDH staining was used to denote the boundary of the cytoplasmic membrane. 100-600 cells per experiment per sample were quantified for cell culture, while all tdT^+^ cells in each image were quantified.

### Immunoprecipitation

Differentiating SVZ NPCs or reprogramming a-pericytes were homogenized and lysed in lysis buffer (25 mM Tris, pH=7.4, 10mM NaCl, 2mM ethylenediaminetetraacetic acid (EDTA), 1mM ethylene glycol tetraacetic acid (EGTA), 0.5% Triton-100, 10% glycerol) containing 1 mM phenylmethylsulfonyl fluoride (PMSF) (Sigma-Aldrich, P7626), 1mM sodium orthovanadate (Sigma-Aldrich, S6508), 20mM sodium fluoride (Fisher, AC201295000), 10 µg/mL aprotinin (Fisher, PI78432) and 10 µg/mL leupeptin (Fisher, PI78436). The extractions were sonicated 3 times with 5 s pulses at 1 min intervals. Then, 200-300 µg protein lysate from each sample was incubated with 30 µL protein A conjugated magnetic beads (Thermo Fisher Scientific, LS10001D) and 6 µg anti-Sox2 antibody at 4ºC overnight. Following that, the magnetic beads were rinsed 3 times with lysis buffer, boiled with sample buffer, and loaded on a 5-15% gradient sodium dodecyl-sulfate polyacrylamide gel electrophoresis (SDS-PAGE) gel. Densitometry was performed using Fiji software.

### Western blot analysis and densitometry

Differentiating SVZ NPCs or reprogramming a-pericytes were lysed in lysis buffer (25 mM Tris, pH=7.4, 10mM NaCl, 2mM EDTA, 1mM EGTA 0.5% Triton-100, 10% glycerol) containing 1 mM PMSF, 1mM sodium orthovanadate, 20mM sodium fluoride, 10 µg/mL aprotinin and 10 µg/mL leupeptin. 10 to 20 µg protein lysates were resolved on a 15% SDS-PAGE gel for assessing H2B acetylation. Densitometry was performed using Fiji software.

### Antibodies

The primary antibodies used for immunohistochemistry: rabbit anti-Sox2 (1:200) (Millipore, AB5603MI), goat anti-DCX (1:200) (Santa Crus, sc-8066), rat anti-CD31 (1:200) (BD Biosciences, 550274), rabbit anti-Col1a1 (1:500) (OriGene Technologies, R1038), rabbit anti-Iba1 (1:1000) (Wake Chemicals, 019-19741), mouse anti-Olig1 (1:200) (EMD Millipore, MAB5540), goat anti-Pdgfrβ (1:500) (R&D systems, AF1042), rabbit anti-Tbx18 (1:100) (Abcam ab86332), and rabbit anti-NG2 (1:500) (EMD Millipore AB5320). The secondary antibodies used for immunohistochemistry: donkey anti-rabbit Alexa Fluor 647 (1:500, Invitrogen, Thermo Fisher Scientific, A21206), donkey anti-goat Alexa Fluor 647 (1:500, Invitrogen, Thermo Fisher Scientific, A-21432), goat anti-rat Alexa Fluor 647 (1:500, Invitrogen, Thermo Fisher Scientific, A-21434), donkey anti-mouse Alexa Fluor 647 (1:500, Invitrogen, Thermo Fisher Scientific, A-31571).

The primary antibodies used for immunocytochemistry: rabbit anti-Sox2 (1:500) (Millipore, MA, AB5603), mouse anti-βIII tubulin (1:500) (Biolegend, 801201), mouse anti-GAPDH (1:500) (Abcam, ab8245), chicken anti-GFP antibody (1:2000) (Abcam, ab13970), rabbit anti-NeuN (1:500) (Invitrogen, PA5-56560), guinea pig anti-Vglut2 (1:500) (Synaptic Systems, 135404), goat anti-Pdgfrβ (1:500) (R&D systems, AF1042), and rabbit anti-NG2 (1:500) (EMD Millipore AB5320). The secondary antibodies used for immunocytochemistry: goat anti-mouse Alexa Fluor 488 (1:500; Thermo Fisher Scientific, A11029), goat anti-chicken Alexa Fluor 488 (1:500; Thermo Fisher Scientific, A11039), donkey anti-rabbit Alexa Fluor 555 (1:500; Thermo Fisher Scientific, A31572), donkey anti-goat Alexa Fluor 647 (1:500, Invitrogen, Thermo Fisher Scientific, A-21432), and goat anti guinea pig Alexa Fluor 647 (1:500, Invitrogen, Thermo Fisher Scientific, A-21450).

The primary antibodies used for Western Blot analysis: rabbit anti-Sox2 (1:500) (Millipore, MA, AB5603), goat anti-Sox2 (1:100) (Santa Cruz Biotechnology, sc-17320), rabbit pan anti-acetylated protein (1:500) (Abcam, ab193), rabbit anti-acetyl-H2BK5 (1:500) (Abcam, ab40886), mouse anti-H2B (1:50000) (Abcam, ab52484) and rabbit anti-CBP (1:500) (Santa Cruz Biotech, sc-583). The secondary antibodies used for Western Blot analysis: Anti-mouse IgG HRP-linked antibody (1:5000; Cell Signaling Technology, 7076S), Anti-rabbit IgG HRP-linked antibody (1:5000; Cell Signaling Technology, 7074S), and anti-goat HRP-linked antibody (1:5000; Santa-Cruz Biotechnology, sc-2354).

### Statistical analysis

Data analysis was performed using GraphPad software for either a two-tailed Student's t-test or one-way ANOVA with Tukey's multiple comparisons as post hoc analysis. Error bars indicate the standard error of the mean (SEM). P-values presented as *P < 0.05, **P < 0.01, ***P < 0.001, and ****P < 0.0001.

## Supplementary Material

Supplementary figures and tables.
