# Supplementary material for: Single-cell profiling of brain pericyte heterogeneity following ischemic stroke unveils distinct pericyte subtype-targeted neural reprogramming potential and its underlying mechanisms
Source: Theranostics. 2024 Sep 23;14(16):6110–37. doi: 10.7150/thno.97165 (PMC11488099; doi:10.7150/thno.97165)
Supplement: Supplementary file 1 — Supplementary figures and tables. [file thnov14p6110s1.pdf]

Supplementary

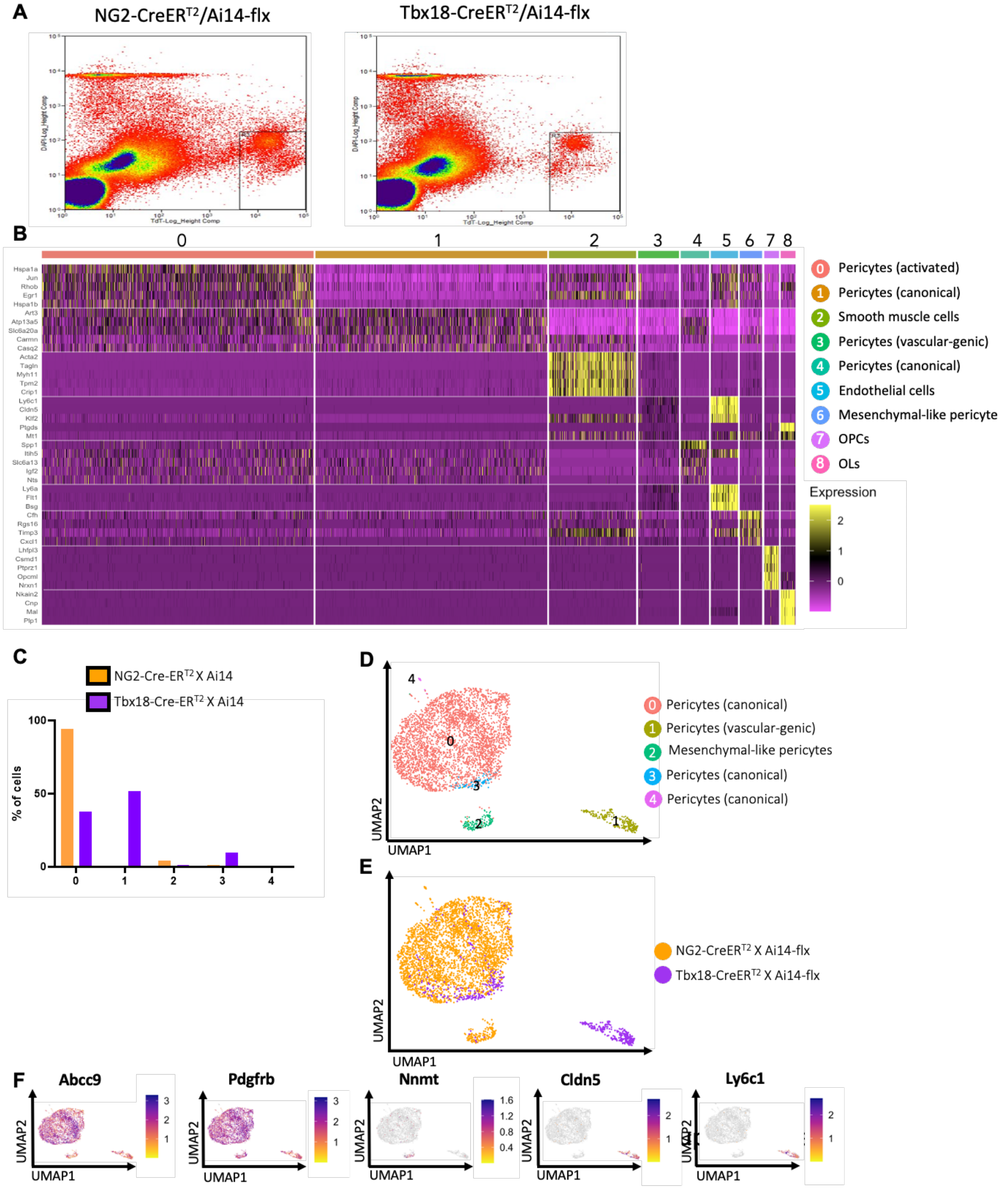

Figure S1: **FAC sorting of *tdT*$^{+}$/DAPI^{-} live cells for scRNA-seq analysis.** (A) Gating strategy for flow cytometry cell sorting of *tdT*$^{+}$/DAPI^{-} live cells from *NG2-CreER^{T2}/Ai14-flx* mice and *Tbx18-CreER^{T2}/Ai14-flx* mice receiving tamoxifen treatment. (B) Heatmap of top five differentially up-regulated genes relative to all other eight clusters from integrated tdT^{+} cells including both naïve NG2-tdT^{+} and naïve Tbx18-tdT^{+} cells. (C) Proportion of cells in naïve NG2-tdT^{+} and naïve Tbx18-tdT^{+} after sub-setting naïve NG2-tdT^{+} and naïve Tbx18-tdT^{+} pericytes clusters. (D) Visualization of cells from naïve NG2-tdT^{+}, naïve Tbx18-tdT^{+} groups after PCA and UMAP, coloured by Seurat

clustering and annotated by cell type. (E) UMAP visualization of NG2-tdT$^+$ (orange) and Tbx18-tdT$^+$ (purple) groups. (F) Visualization of the total cell population after PCA and UMAP, coloured by expression of key marker genes (*Abcc9*, *Pdgfrβ*, *Nnmt*, *Cldn5*, and *Ly6c1*).

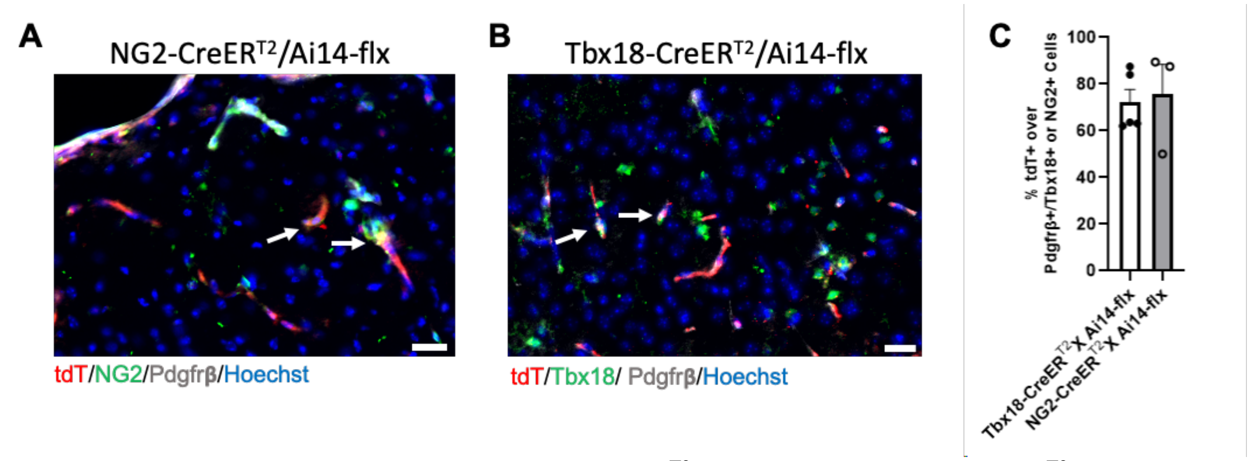

Figure S2: **Recombination rate for *Tbx18-CreER^{T2}/Ai14-flx* and *NG2-CreER^{T2}/Ai14-flx*.** (A-C) Representative images and quantifications of the proportion of tdT^{+} (red) cells over total Pdgfrβ (grey) cells co-labelled with either (A) NG2 (green) or (B) Tbx18 (green), and counterstained for Hoechst (blue). Scale bar = 25 µm. n = 3-5.

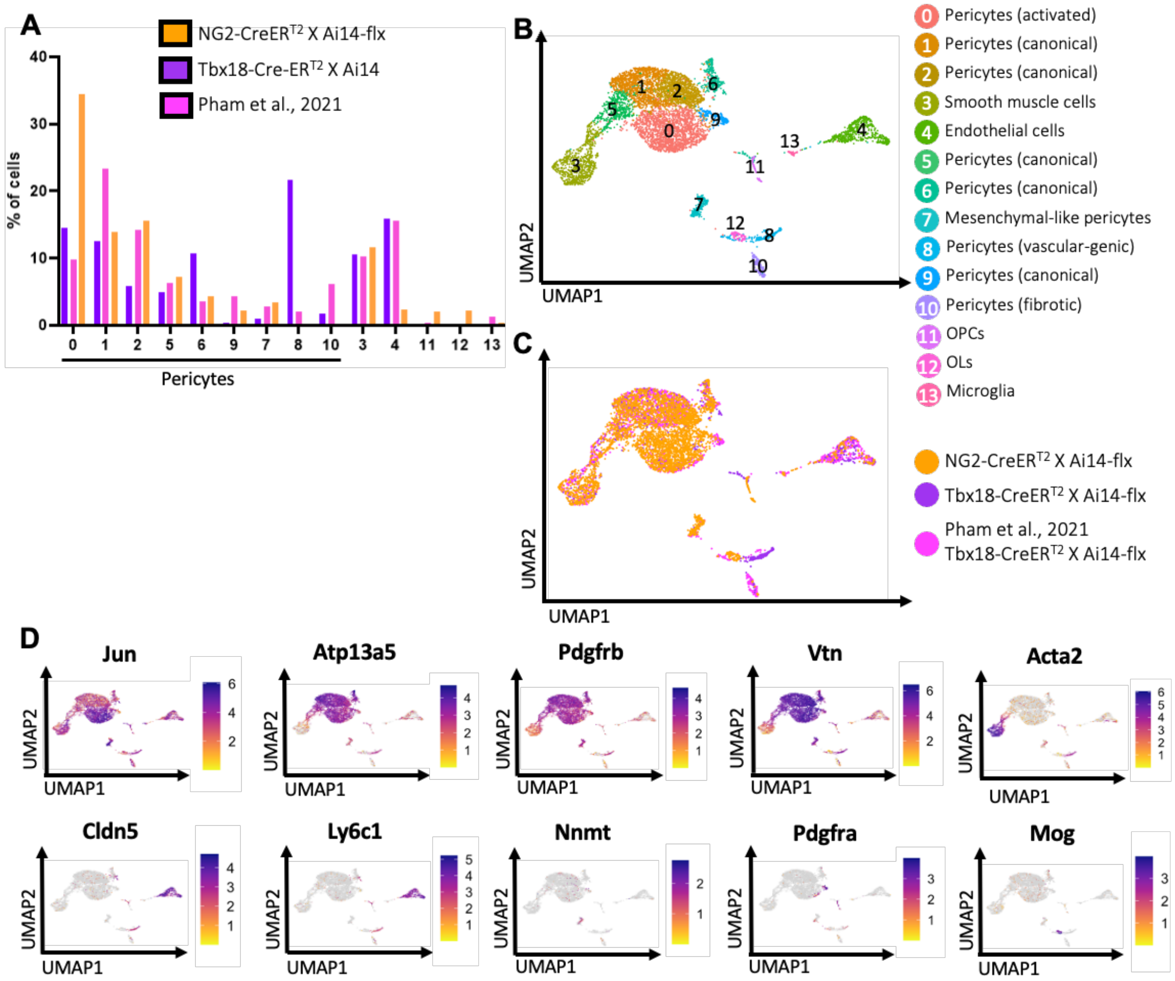

Figure S3. **Utilization of a publicly available *Tbx18-tdT*⁺ dataset obtained from Pham *et al.*, 2021 for confirmation of the naïve *Tbx18-tdT*⁺ phenotype**. (A) Proportion of cells in naïve NG2-tdT⁺, naïve Tbx18-tdT⁺, and sham Tbx18-tdT⁺ obtained from Pham et al., 2021. (B) Visualization of cells from naïve NG2-tdT⁺, naïve Tbx18-tdT⁺, and sham Tbx18-tdT⁺ groups after PCA and UMAP, coloured by Seurat clustering and annotated by cell type. (C) UMAP visualization of naïve NG2-tdT⁺ (orange), naïve Tbx18-tdT⁺ (purple), and sham Tbx18-tdT⁺ (pink) groups. (D) Visualization of the total cell population after PCA and UMAP, coloured by expression of key marker genes (*Jun*, *Atp13a5*, *Pdgfrβ*, *Vtn*, *Acta2*, *Cldn5*, *Ly6c1*, *Nnmt*, *Pdgfrα*, and *Mog*).

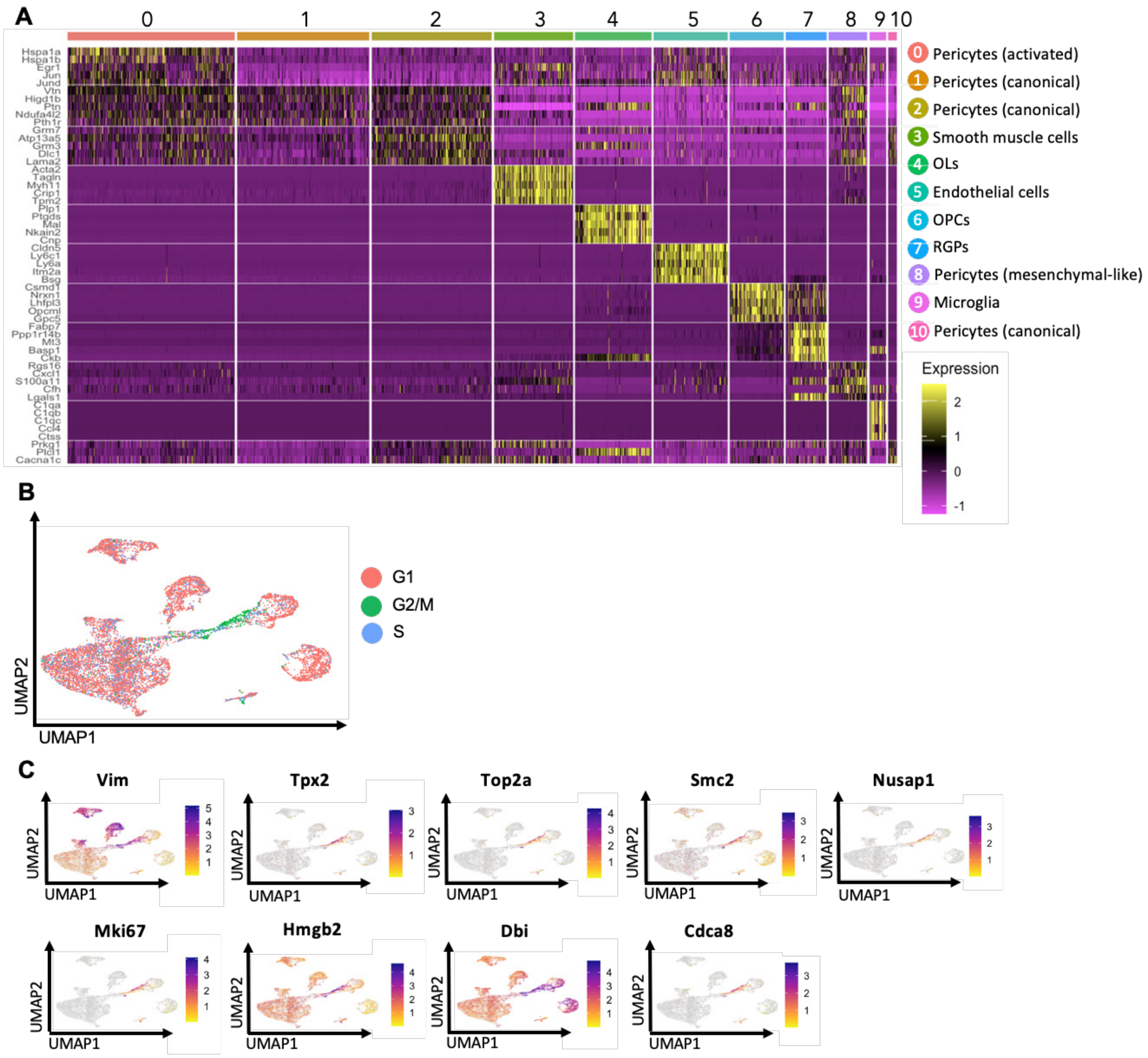

Figure S4: **NG2-tdT$^+$ cells** (A) Heatmap of top five differentially up-regulated genes relative to all other ten clusters from integrated tdT$^+$ cells including naïve NG2-tdT$^+$, physical injury NG2-tdT$^+$ and ischemic injury NG2-tdT$^+$ cells. (B) Visualization of the total NG2-tdT$^+$ cell population after PCA and UMAP, coloured by cell cycle phase. (C) Visualization of the total NG2-tdT$^+$ cell population after PCA and UMAP, coloured by RGP marker genes (*Vim, Tpx2, Top2a, Smc2, Nusap1, Mik67, Hmgb2, Dbi,* and *Cdca8*).

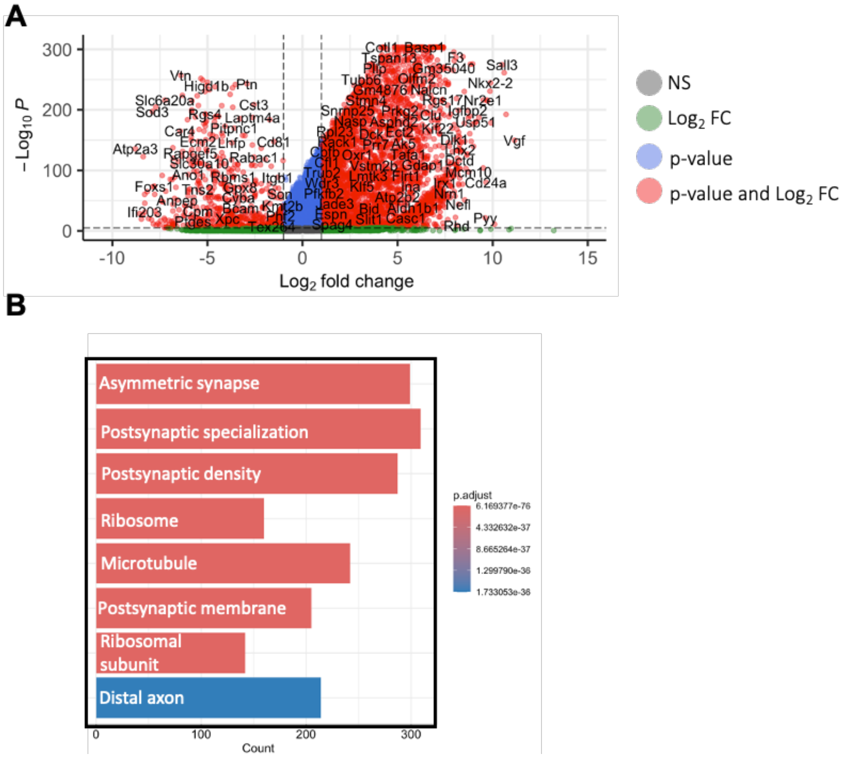

Figure S5: **Differentially expressed genes and downstream GO analysis of cluster 7 (RGPs) compared to cluster 1 (canonical pericytes).** (A) Volcano plot of differentially expressed genes in cluster 7 (RGPs) compared to cluster 1 (canonical pericytes). Discriminated based on p-value adjusted and log2 fold-change. Log2 fold-change > 2 and p-value adjusted < $10e^{-6}$. (B) GO enrichment (cellular component) results of upregulated differentially expressed genes in cluster 7 (RGPs) compared to cluster 1 (canonical pericytes). Log2 fold-change > 0.25 and p-value adjusted < 0.05.

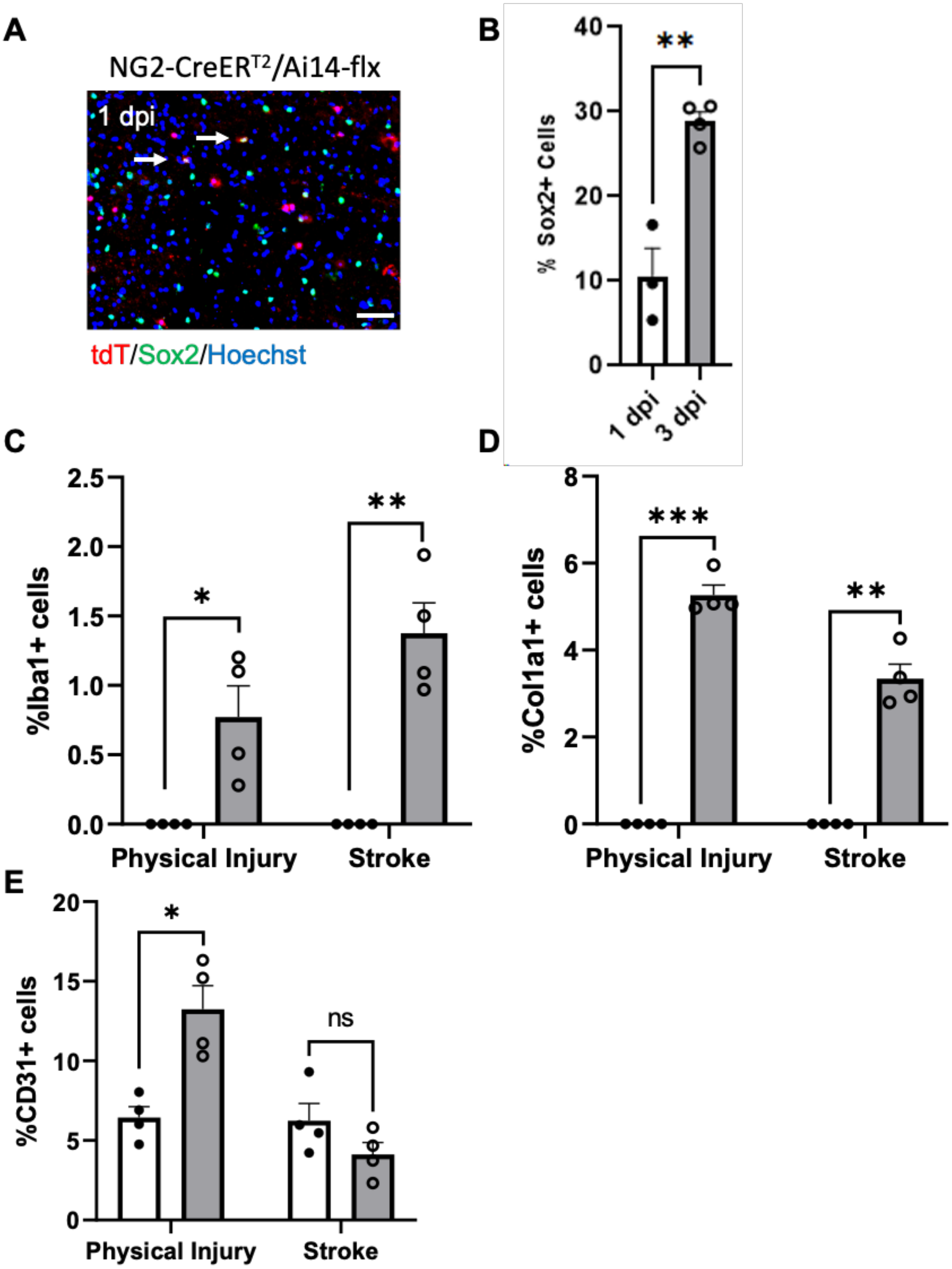

Figure S6: **Images and quantitative analysis of the proportion of Sox2^{+}/tdT^{+} i-NSCs over total NG2-tdT^{+} cells in the injured cortex sections from mice receiving ET-1/L-NAME (stroke) injections, collected 1 and 3 days post-injury (A-B)**, immunostained for Sox2 (green) and tdT (red), and counterstained for Hoechst (blue). White arrows represent Sox2^{+}/tdT^{+} cells**.** Scale bar: 500 µm. n=3-4 animals/group. Student t-test, **P < 0.01. **Immunohistochemical**

analysis of NG2-tdT$^+$ cells that express Iba1$^+$ microglia (C), Col1A1$^+$ fibroblasts (D) and CD31$^+$ micro-vessels (E). (Smaller scale of Y-axis was presented compared to the graphs shown in main Figure 3).

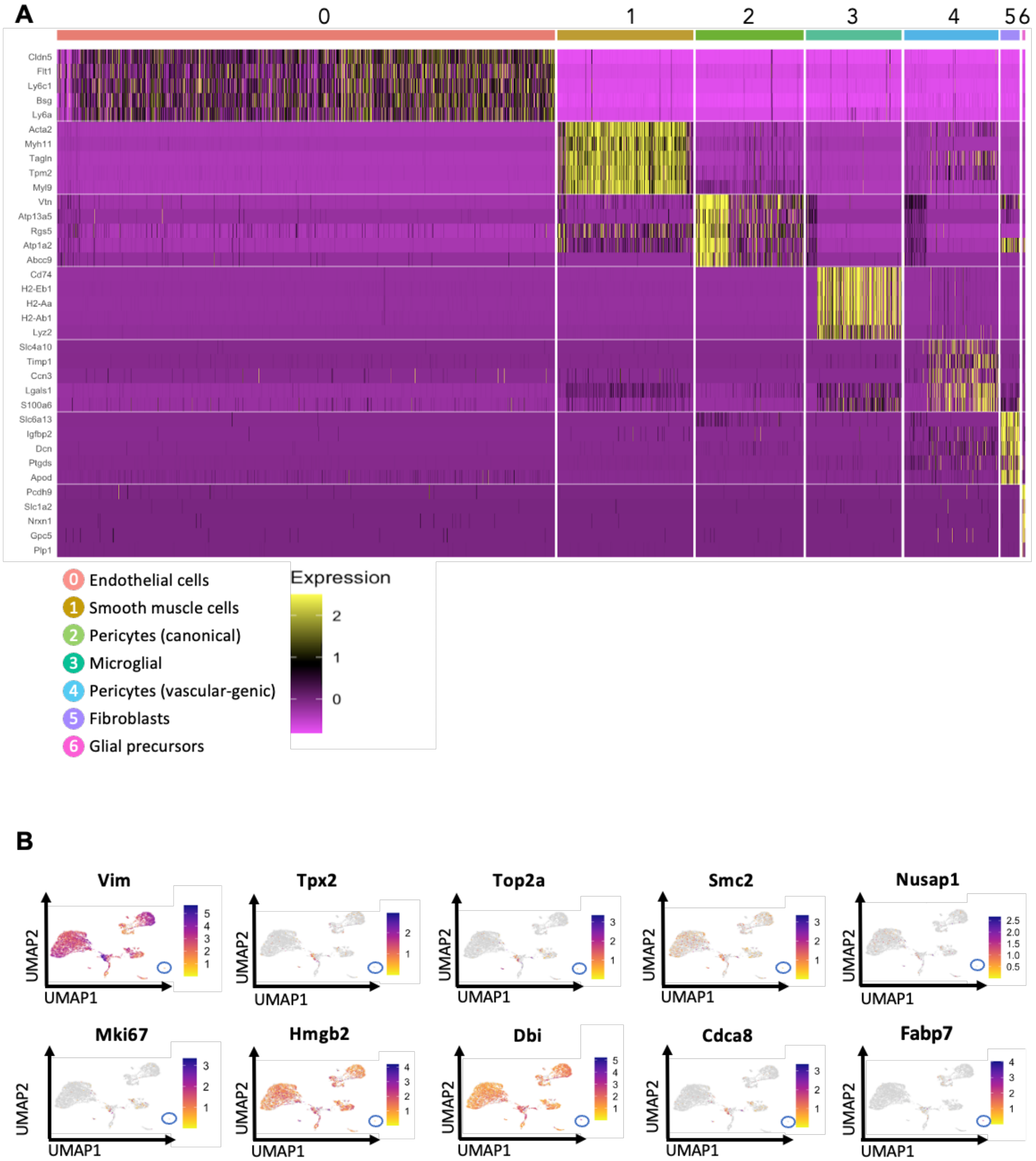

Figure S7: **Tbx18-tdT$^+$ cells.** (A) Heatmap of top five differentially up-regulated genes relative to all other six clusters from integrated tdT$^+$ cells including naïve Tbx18-tdT$^+$, physical injury Tbx18-tdT$^+$ and ischemic injury Tbx18-tdT$^+$ cells. (B) Visualization of the total Tbx18-tdT$^+$ cell population after PCA and UMAP, coloured by RGP marker genes (*Vim, Tpx2, Top2a, Smc2, Nusap1, Mik67, Hmgb2, Dbi, Cdca8,* and *Fabp7*).

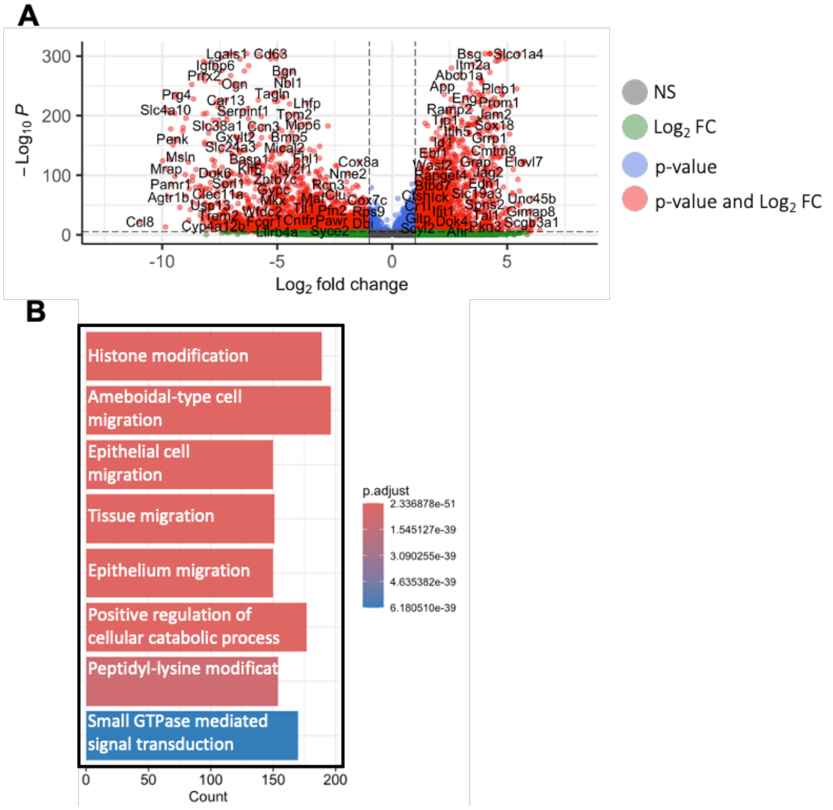

Figure S8: **Differentially expressed genes and downstream GO analysis of cluster 0 (endothelial cells) compared to cluster 4 (vascular-genic pericytes).** (A) Volcano plot of differentially expressed genes in cluster 0 (endothelial cells) compared to cluster 4 (vascular-genic pericytes). Discriminated based on p-value adjusted and log2 fold-change. Log2 fold-change > 2 and p-value adjusted < $10e^{-6}$. (B) GO enrichment (biological process) results of upregulated differentially expressed genes in cluster 0 (endothelial cells) compared to cluster 4 (vascular-genic pericytes). Log2 fold-change > 0.25 and p-value adjusted < 0.05.

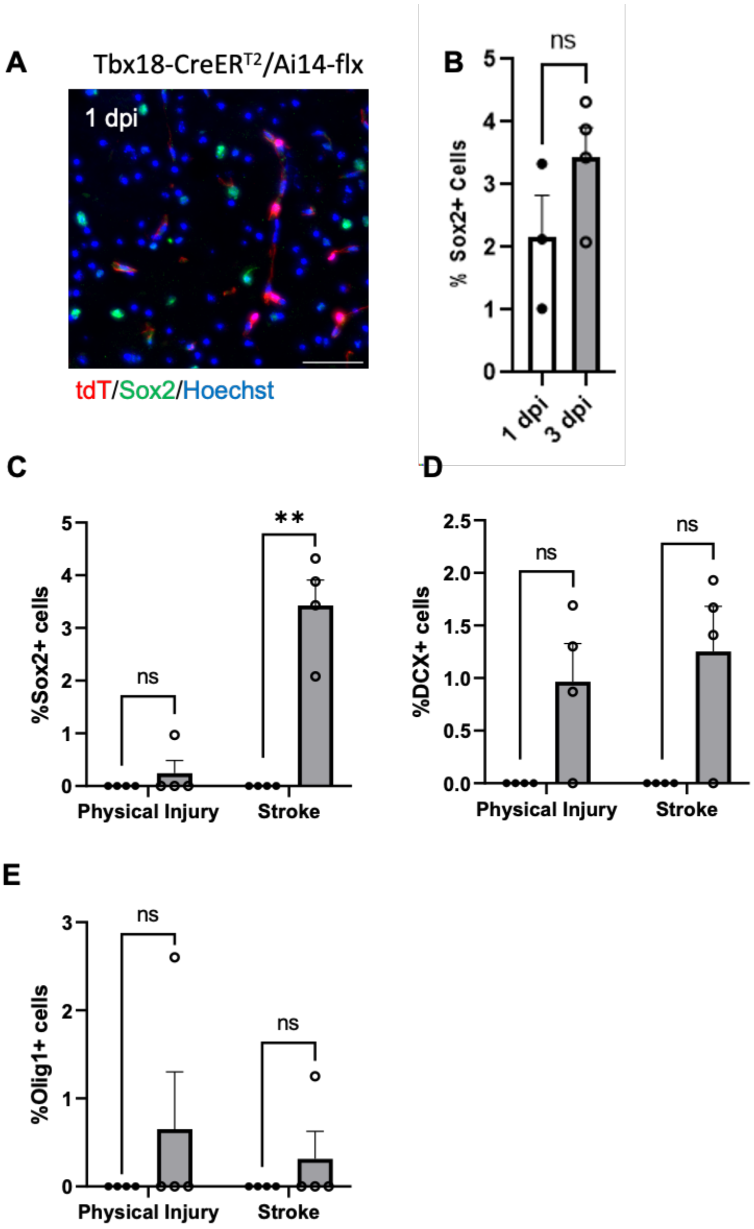

Figure S9: **Images and quantitative analysis of the proportion of $Sox2^{+}/tdT^{+}$ i-NSCs over total Tbx18-tdT^{+} cells in the injured cortex sections from mice receiving ET-1/L-NAME (stroke) injections, collected 1 and 3 days post-injury (A-B)**, immunostained for Sox2 (green) and tdT (red), and counterstained for Hoechst (blue). Scale bar: 100 µm. n=3-4 animals/group. Student t-test, $^{**}P < 0.01$. **Immunohistochemical analysis of Tbx18-tdT^{+} cells that express $Sox2^{+}$ i-NSCs (C), DCX^{+} neuroblasts (D) and $Olig1^{+}$ micro-vessels (E).** (Smaller scale of Y-axis was presented compared to the graphs shown in main Figure 5).

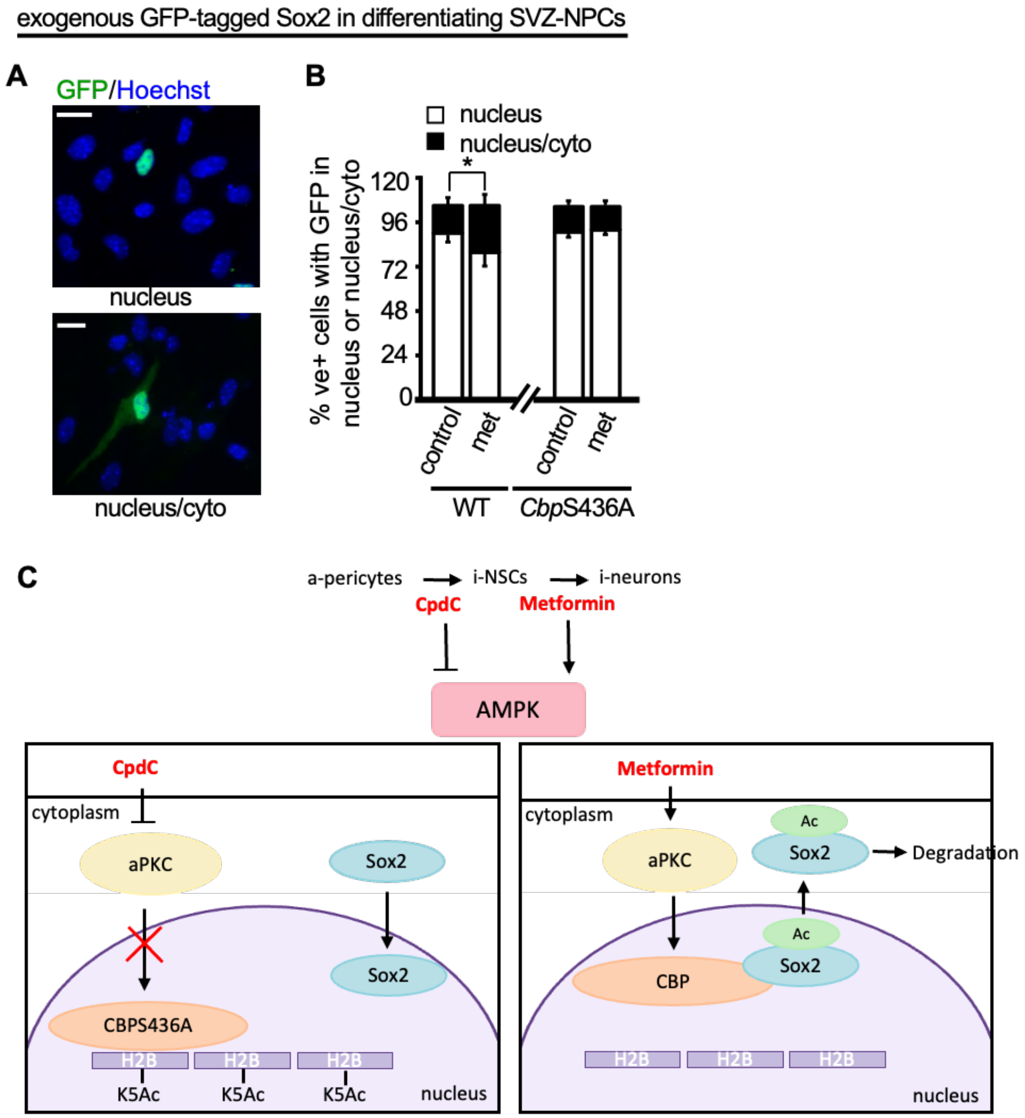

Figure S10: **Exogenous GFP-tagged Sox2 nuclear/cyto trafficking in differentiating SVZ NPCs.** (A-B) Representative images and analysis of the percentage of differentiating WT and *Cbp*S436A SVZ NPCs transfected with GFP-fused hSox2, expressing GFP in either nucleus alone or both nuclear and cytoplasmic localization, in the absence and presence of metformin for 3 days. These NPCs were treated with MG132 (1 μM) for 16 h prior to fixation. Scale bar: 20 μm. *P < 0.05; n=4 animals/group. (C) Proposed model of i-NSC generation from a-pericytes via treatment with CpdC to block the aPCK-CBP pathway and promote Sox2 trafficking into the nucleus by shifting acetylation away from Sox2 towards H2BK5. Proposed model of i-neuron generation from i-NSCs via treatment with metformin to activate the aPCK-CBP pathway to promote Sox2 acetylation and trafficking out of the nucleus by shifting the acetylation away from H2B towards Sox2.

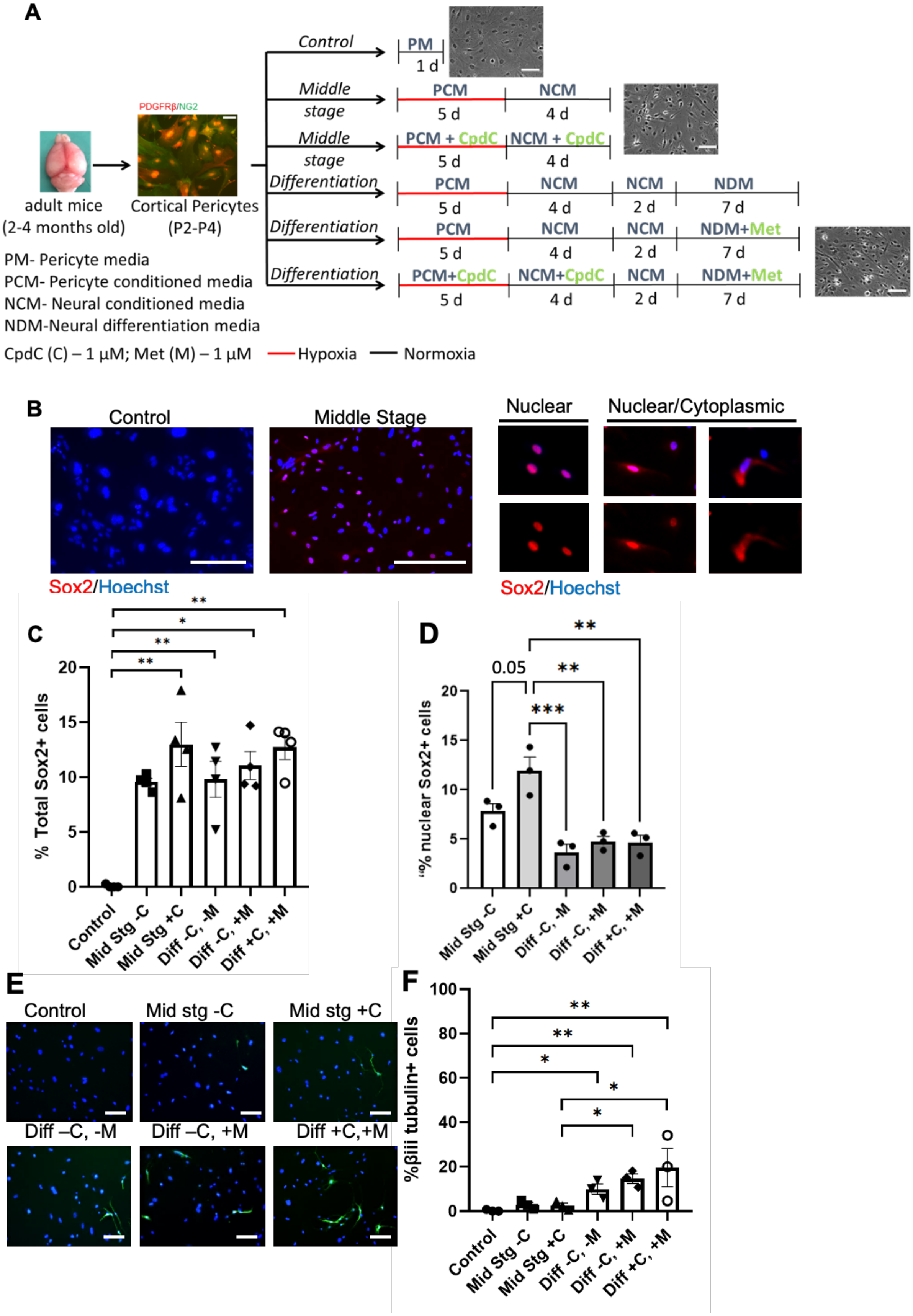
A
PDGFRβ/NG2
adult mice
(2-4 months old)
Cortical Pericytes
(P2-P4)
Control
PM
1 d
Middle stage
PCM
NCM
5 d
4 d
Middle stage
PCM + CpdC
NCM + CpdC
5 d
4 d
Differentiation
PCM
NCM
NCM
NDM
5 d
4 d
2 d
7 d
Differentiation
PCM
NCM
NCM
NDM+Met
5 d
4 d
2 d
7 d
Differentiation
PCM+CpdC
NCM+CpdC
NCM
NDM+Met
5 d
4 d
2 d
7 d
PM- Pericyte media
PCM- Pericyte conditioned media
NCM- Neural conditioned media
NDM-Neural differentiation media
CpdC (C) – 1 µM; Met (M) – 1 µM
Hypoxia
Normoxia
B
Control
Middle Stage
Nuclear
Nuclear/Cytoplasmic
Sox2/Hoechst
Sox2/Hoechst
C
% Total Sox2+ cells
Control
Mid Stg -C
Mid Stg +C
Diff -C, -M
Diff -C, +M
Diff +C, +M
D
% nuclear Sox2+ cells
0.05
Mid Stg -C
Mid Stg +C
Diff -C, -M
Diff -C, +M
Diff +C, +M
E
Control
Mid stg -C
Mid stg +C
Diff –C, -M
Diff –C, +M
Diff +C,+M
F
%βiii tubulin+ cells
Control
Mid Stg -C
Mid Stg +C
Diff -C, -M
Diff -C, +M
Diff +C, +M

Figure S11. **Sequential treatment of CpdC and metformin facilitates reprogramming /differentiation of NG2$^+$ murine cortical pericyte to generate neurons in culture.** (A) Experimental timeline of cultured murine cortical pericytes undergoing different conditions for neural reprogramming/differentiation. (B) Images of cultured murine cortical pericytes receiving hypoxia treatment combined with PCM and NCM for neural reprogramming at the middle stage, immunostained for Sox2 (red), counterstained with Hoechst (blue). Sox2 expression is denoted as nuclear and nuclear/cytoplasmic localization. (C) Quantification of the proportion of total Sox2$^+$ i-NSCs over total live cells from various conditions including control, middle stage, middle stage + CpdC, differentiation stage, differentiation stage + Met, and middle stage with CpdC treatment + differentiation with met treatment. (D) Quantification of the proportion of nuclear Sox2$^+$ i-NSCs from various conditions as shown in A and C. (E-F) Representative images and quantification of the proportion of βIII tubulin$^+$ (green) neurons over total live cells from various conditions as shown in A and C. Scale bar: 200 µm (B); 100 µm (E). n = 3-4 animals/group; One-way ANOVA, *$P < 0.05$, ** $P < 0.01$, *** $P < 0.001$, **** $P < 0.0001$.

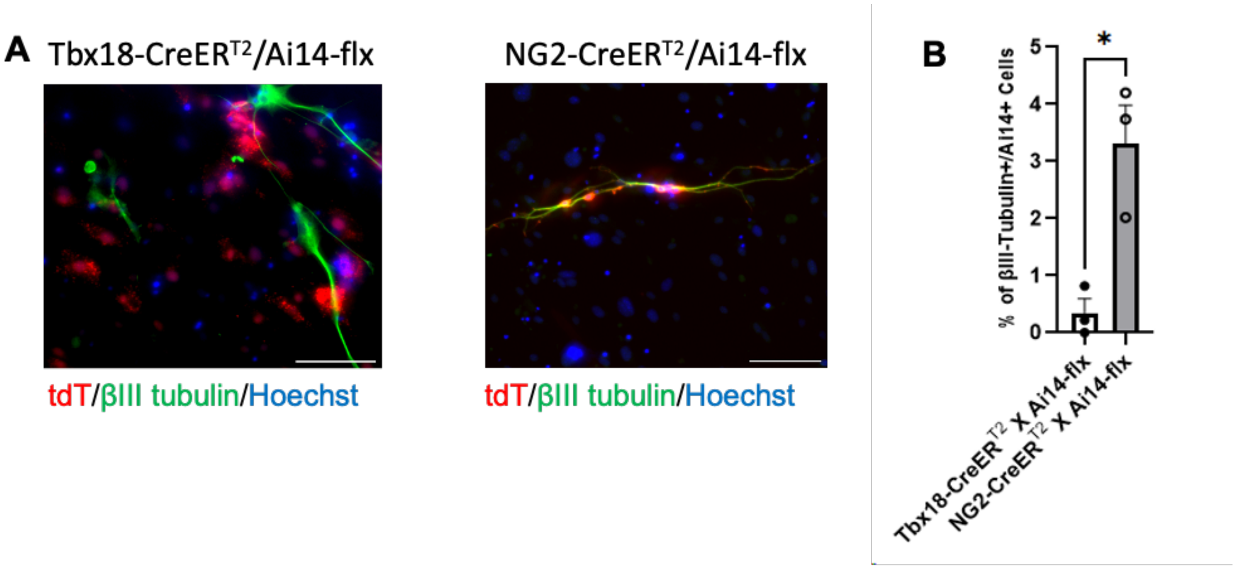

Figure S12: **Immunohistochemical analysis of Tbx18-tdT^{+} and NG2-tdT^{+} pericytes in vitro. (A-B)** Images and quantitative analysis of the proportion of βIII tubulin^{+}/tdT^{+} i-neurons, immunostained for βIII tubulin (green) and tdT (red), and counterstained for Hoechst (blue). Scale bar: 100 µm. n=3 animals/group. Student t-test, *P < 0.05.

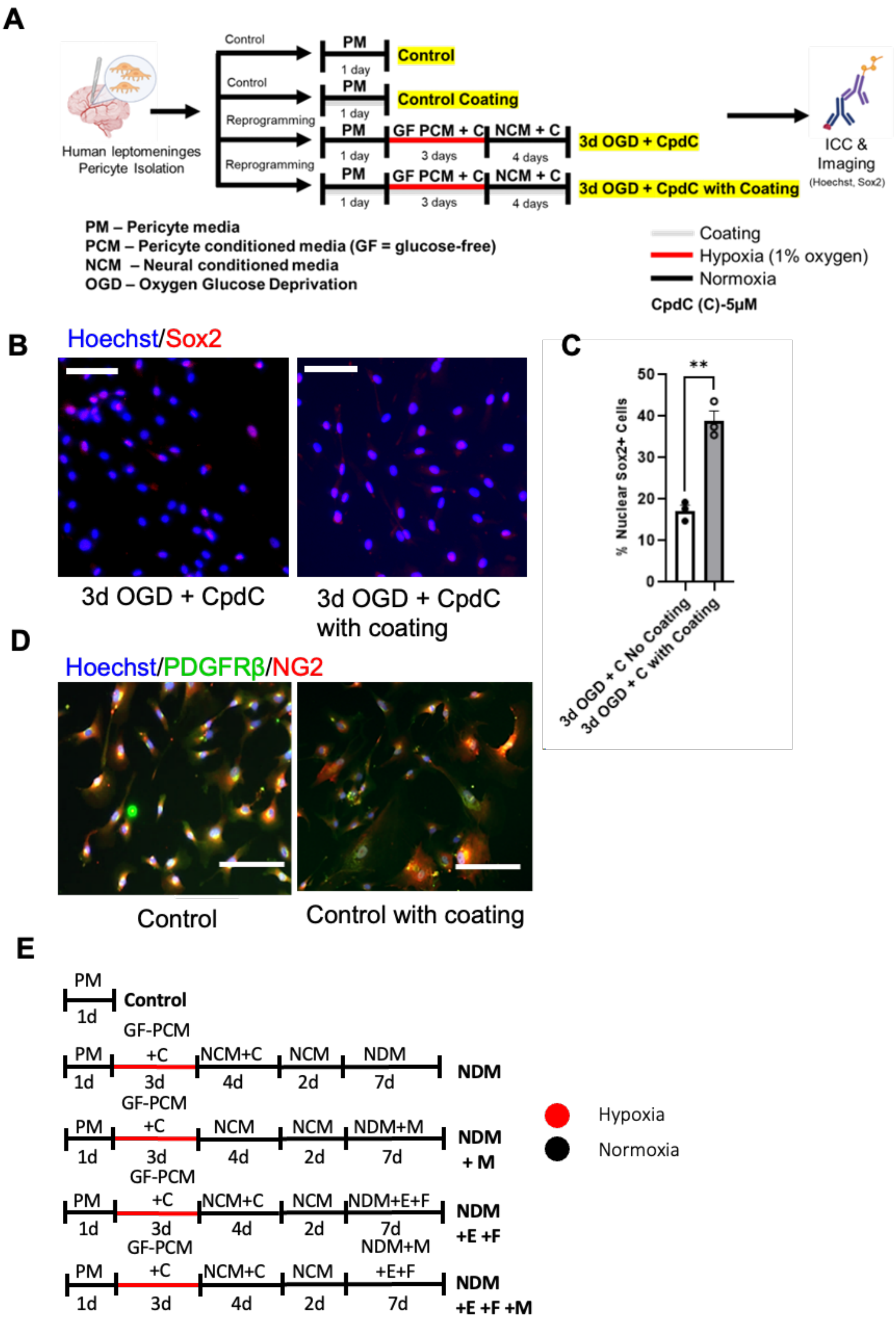
A
Human leptomeninges Pericyte Isolation
Control
PM
1 day
Control
Control
PM
1 day
Control Coating
Reprogramming
PM
1 day
GF PCM + C
3 days
NCM + C
4 days
3d OGD + CpdC
Reprogramming
PM
1 day
GF PCM + C
3 days
NCM + C
4 days
3d OGD + CpdC with Coating
ICC & Imaging
(Hoechst, Sox2)
PM – Pericyte media
PCM – Pericyte conditioned media (GF = glucose-free)
NCM – Neural conditioned media
OGD – Oxygen Glucose Deprivation
Coating
Hypoxia (1% oxygen)
Normoxia
CpdC (C)-5μM
B
Hoechst/Sox2
3d OGD + CpdC
3d OGD + CpdC with coating
C
% Nuclear Sox2+ Cells
50
40
30
20
10
0
**
3d OGD + C No Coating
3d OGD + C with Coating
D
Hoechst/PDGFRβ/NG2
Control
Control with coating
E
PM
1d
Control
PM
1d
GF-PCM +C
3d
NCM+C
4d
NCM
2d
NDM
7d
NDM
PM
1d
GF-PCM +C
3d
NCM
4d
NCM
2d
NDM+M
7d
NDM + M
PM
1d
GF-PCM +C
3d
NCM+C
4d
NCM
2d
NDM+E+F
7d
NDM +E +F
PM
1d
GF-PCM +C
3d
NCM+C
4d
NCM
2d
NDM+M +E+F
7d
NDM +E +F +M
Hypoxia
Normoxia

Figure S13. **Poly-L-ornithine and laminin coating enhances human pericyte neural reprogramming to produce i-NSCs.** (A) Experimental timeline of cultured human pericyte isolated from human leptomeningeal tissues. Experimental groups are highlighted in yellow. (B-C) Representative images and quantification of the proportion of nuclear $Sox2^{+}$ (red) i-NSCs over total live cells from 3d OGD + CpdC and 3d OGD + CpdC with coating conditions. (D) Image of cultured human pericytes in the absence and presence of coating materials, immunostained for NG2 (red) and Pdgfrβ (green), and counterstained for Hoechst (blue). Scale bar: 100 µm. n = 3 donor tissues/group; a two-tailed student t-test, ** $P < 0.01$. (E) Experimental timeline of cultured human pericytes-derived i-NSCs undergoing different conditions for neuronal differentiation.

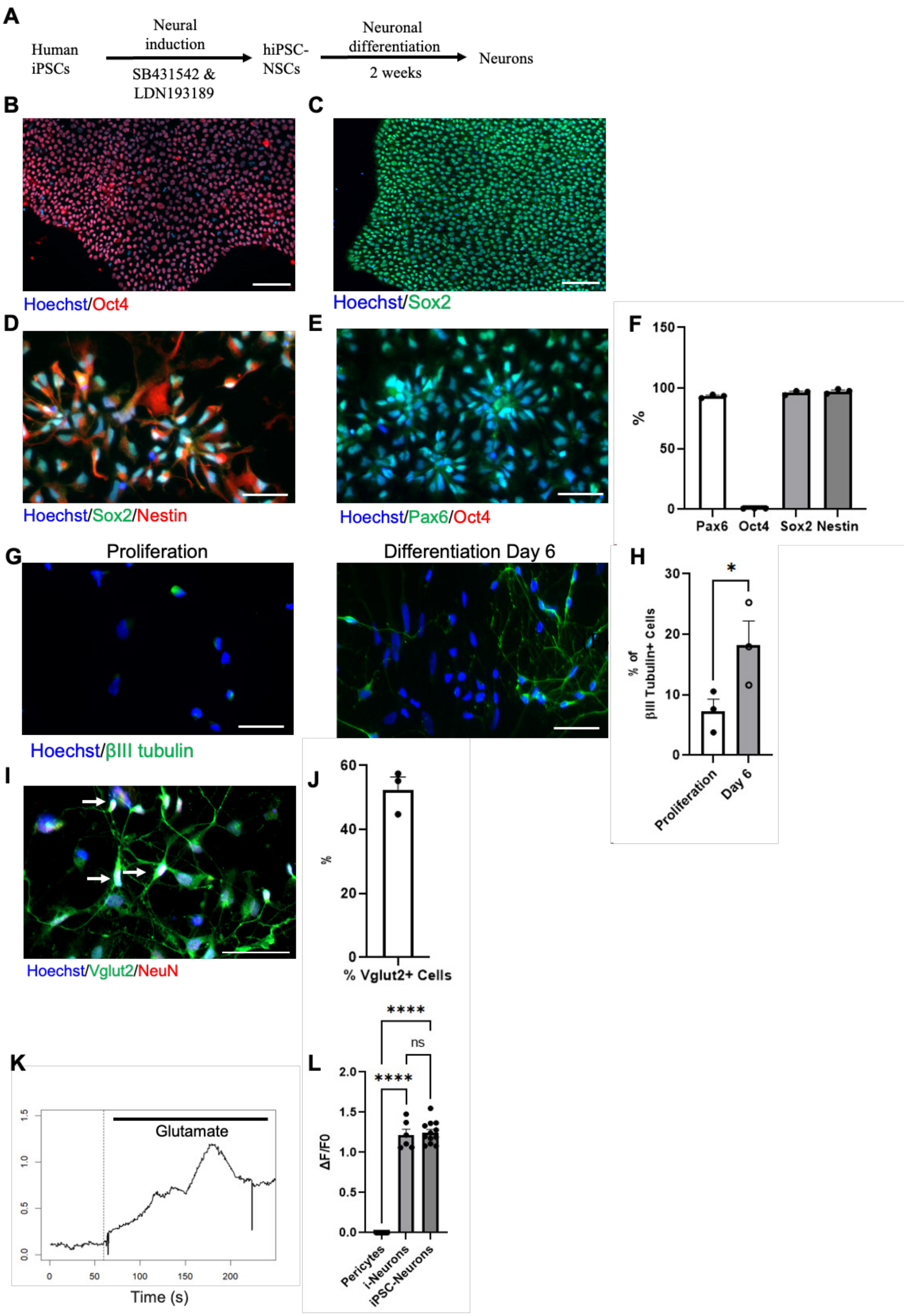

A
Human iPSCs
Neural induction
SB431542 & LDN193189
hiPSC-NSCs
Neuronal differentiation
2 weeks
Neurons
B
Hoechst/Oct4
C
Hoechst/Sox2
D
Hoechst/Sox2/Nestin
E
Hoechst/Pax6/Oct4
F
%
Pax6
Oct4
Sox2
Nestin
G
Proliferation
Differentiation Day 6
Hoechst/βIII tubulin
H
% of βIII Tubulin+ Cells
Proliferation
Day 6
I
Hoechst/Vglut2/NeuN
J
%
% Vglut2+ Cells
K
Glutamate
Time (s)
L
ΔF/F0
****
ns
Pericytes
i-Neurons
iPSC-Neurons

Figure S14. **The genesis of human iPSC-derived neurons.** (A) Schematic workflow of culturing hiPSC-derived neural stem cells (NSCs), which underwent neuronal differentiation to produce newborn neurons. (B-C) Image of human iPSCs immunostained for (B) Oct4 (red) and (C) Sox2 (green), counterstained with Hoechst (blue). Scale bar: 100 µm. (D-F) Representative images and quantification of hiPSC-NSCs immunostained for (D) Sox2 (green) and Nestin (red) or (E) Pax6 (green) and Oct4 (red), counterstained with Hoechst (blue). Scale bar: 50 µm. n=3 independent experiments. (G-H) Representative images and quantification of neurons at the proliferation stage and differentiation on day 6, immunostained for βIII-tubulin (green) and counterstained with Hoechst (blue). Scale bar: 50 µm. n=3 independent experiments. Student t-test, $*P < 0.05$. (I-J) Representative images and quantification of the proportion of $Vglut2^+$ (green) and $NeuN^+$ (red) in iPSC-derived neurons over total live cells. Student t-test, $* P < 0.05$. n = 3-4 independent experiments; Scale bar:50 µm. Arrows denote $Vglut2^+/NeuN^+$ double labeled neurons. (K) Representative fluorescence traces of Fluo 4 AM before and after glutamate (10 µM) addition at 60 s from iPSC-Neurons. (L). Quantification of amplitude of spikes in response to glutamate from human pericyte-derived i-neurons and hiPSC-Neurons. n = 6-12 cells/group; One-way ANOVA, $**** P < 0.0001$.

Table S1. Annotations, abundance, and marker genes of each cell cluster used for UMAP visualizations of naïve *NG2-tdT*+ cells and naïve *Tbx18-tdT*+ cells.

Cluster #	**Cluster name**	**Markers**	**% in naïve *NG2-tdT*+**	**% in naïve *Tbx18-tdT*+**
0	Pericytes (activated)	*Jun*+, *Abcc9*+, *Pdgfrβ*+, *Vtn*+, and *Atp13a5*+	39.4101254	20.4081633
1	Pericytes (canonical)	*Abcc9*+, *Pdgfrβ*+, *Vtn*+, and *Atp13a5*+	33.6274965	17.3469388
2	Smooth muscle cells	*Acta2*+, *Tagln*+, and *Myh11*+	11.9600557	10.787172
3	Pericytes (vascular-genic)	*Cldn5*+, *Ly6c1*+, *Abcc9*+, *Pdgfrβ*+, and *Vtn*+	0.6967023	35.7142857
4	Pericytes (canonical)	*Spp1*+, *Atp13a5*+, *Abcc9*+, *Pdgfrβ*+, and *Vtn*+	4.08732	2.1865889
5	Endothelial cells	*Cldn5*+, *Ly6c1*+, and *Klf2*+	2.3455643	11.9533528
6	Mesenchymal-like pericytes	*Nnmt*+, *Cfh*+, *RGS16*+, *Abcc9*+, *Pdgfrβ*+, and *Vtn*+	3.3441709	1.0204082
7	OPCs	*Pdgfrα*+ and *Ptprz1*+	2.2991175	0.1457726
8	OLs	*Mog*+, *Cnp*+, and *Plp1*+	2.2294473	0.4373178

Table S2. Annotations, abundance, and marker genes of each cell cluster used for UMAP visualizations of *NG2-tdT*⁺ cells and *Tbx18-tdT*⁺ cells.

Group	Cluster #	Cluster name	Markers	% in naïve	% in physical injury	% in ischemic injury
NG2-tdT$^+$ *cells*	0	Pericytes (activated)	*Jun*$^+$, *Abcc9*$^+$, *Pdgfrβ*$^+$, *Vtn*$^+$, and *Atp13a5*$^+$	29.7259638	14.8653921	14.026771
NG2-tdT$^+$ *cells*	1	Pericytes (canonical)	*Abcc9*$^+$, *Pdgfrβ*$^+$, *Vtn*$^+$, and *Atp13a5*$^+$	25.2206224	12.4073352	8.616843
NG2-tdT$^+$ *cells*	2	Pericytes (canonical)	*Abcc9*$^+$, *Pdgfrβ*$^+$, *Vtn*$^+$, and *Atp13a5*$^+$	20.3669299	15.8798283	7.612939
NG2-tdT$^+$ *cells*	3	Smooth muscle cells	*Acta2*$^+$, *Tagln*$^+$, and *Myh11*$^+$	11.6813748	9.4420601	7.724484
NG2-tdT$^+$ *cells*	4	OL s	*Mog*$^+$, *Cnp*$^+$, and *Plp1*$^+$	2.3920111	11.4709325	16.508645
NG2-tdT$^+$ *cells*	5	Endothelial cells	*Cldn5*$^+$, *Ly6c1*$^+$, and *Ly6a*$^+$	2.3455643	15.762778	12.63246
NG2-tdT$^+$ *cells*	6	OPCs	*Pdgfrα*$^+$	2.3223409	11.7440499	8.19855
NG2-tdT$^+$ *cells*	7	RGPs	*Fabp7*$^+$ and *Sox2*$^+$	0.394798	2.9652751	12.214166
NG2-tdT$^+$ *cells*	8	Mesenchymal-like pericytes	*Nnmt*$^+$, *Cfh*$^+$, *Rgs16*$^+$, *Abcc9*$^+$, *Pdgfrβ*$^+$, and *Vtn*$^+$	4.1105434	3.160359	6.692694
NG2-tdT$^+$ *cells*	9	Microglia	*C1qa*$^+$, *C1qb*$^+$, *C1qc*$^+$, and Ccl4$^+$	0.4180214	1.404604	4.573341
NG2-tdT$^+$ *cells*	10	Pericytes (canonical)	Abcc9$^+$, Pdgfrβ$^+$, Vtn$^+$, and Atp13a5$^+$	1.02183	0.8973859	1.199108
Tbx18-tdT$^+$ *cells*	0	Endothelial cells	Cldn5$^+$, Flt1$^+$, Ly6c1$^+$, and Cd34$^+$	23.177843	46.5019506	72.691744
Tbx18-tdT$^+$ *cells*	1	Smooth muscle cells	*Acta2*$^+$, *Myh11*$^+$, and *Tagln*$^+$	9.183673	21.5084525	2.296043

Tbx18-tdT^{+} *cells*	2	Pericytes (canonical)	*Vtn*$^{+}$, *Abcc9*$^{+}$, *Atp13a5*$^{+}$, and *Rgs5*$^{+}$	32.944606	11.2613784	4.2012702
Tbx18-tdT^{+} *cells*	3	Microglia	*Cd74*$^{+}$ and *C1qa*$^{+}$	11.078717	8.2704811	12.9457743
Tbx18-tdT^{+} *cells*	4	Pericytes (vascular-genic)	*Pdgfrβ*$^{+}$, *Spp1*$^{+}$, *Vtn*$^{+}$, *Acta2*$^{+}$, and *Cldn5*$^{+}$	22.886297	9.076723	6.9858329
Tbx18-tdT^{+} *cells*	5	Fibroblasts	*Col1a1*$^{+}$, *Col15a1*$^{+}$, and *Dcn*$^{+}$	0.728863	3.0169051	0.4885198
Tbx18-tdT^{+} *cells*	6	Glial precursors	*Pcdh1*$^{+}$. *Slc1a2*$^{+}$, and *Sox2*$^{+}$	0	0.3641092	0.3908158
